# Integrating Network Pharmacology, Transcriptome and Artificial Intelligence for Investigating Into the Effect and Mechanism of Ning Fei Ping Xue Decoction Against the Acute Respiratory Distress Syndrome

**DOI:** 10.3389/fphar.2021.731377

**Published:** 2021-11-03

**Authors:** Xiaoxiao Lu, Wentao Ma, Baofeng Fan, Peng Li, Jing Gao, Qiuhong Liu, Chunling Hu, Yong Li, Mengying Yao, Hanbing Ning, Lihua Xing

**Affiliations:** ^1^ Department of Respiratory and Critical Care Medicine, The First Affiliated Hospital of Zhengzhou University, Zhengzhou, China; ^2^ Air Force General Hospital PLA, Beijing, China; ^3^ Department of Basic Sciences, Shanxi Agricultural University, Taigu, China; ^4^ Department of Digestive Diseases, The First Affiliated Hospital of Zhengzhou University, Zhengzhou, China

**Keywords:** Ning Fei Ping Xue decoction, acute respiratory distress syndrome, network pharmacology, transcriptome analysis, artificial intelligence analysis, inflammatory responses

## Abstract

Acute respiratory distress syndrome (ARDS) is a high-mortality disease and lacks effective pharmacotherapy. A traditional Chinese medicine (TCM) formula, Ning Fei Ping Xue (NFPX) decoction, was demonstrated to play a critical role in alleviating inflammatory responses of the lung. However, its therapeutic effectiveness in ARDS and active compounds, targets, and molecular mechanisms remain to be elucidated. The present study investigates the effects of NFPX decoction on ARDS mice induced by lipopolysaccharides (LPS). The results revealed that NFPX alleviated lung edema evaluated by lung ultrasound, decreased lung wet/Dry ratio, the total cell numbers of bronchoalveolar lavage fluid (BALF), and IL-1β, IL-6, and TNF-α levels in BALF and serum, and ameliorated lung pathology in a dose-dependent manner. Subsequently, UPLC-HRMS was performed to establish the compounds of NFPX. A total of 150 compounds in NFPX were characterized. Moreover, integrating network pharmacology approach and transcriptional profiling of lung tissues were performed to predict the underlying mechanism. 37 active components and 77 targets were screened out, and a herbs-compounds-targets network was constructed. Differentially expressed genes (DEGs) were identified from LPS-treated mice compared with LPS combined with NFPX mice. GO, KEGG, and artificial intelligence analysis indicated that NFPX might act on various drug targets. At last, potential targets, HRAS, SMAD4, and AMPK, were validated by qRT-PCR in ARDS murine model. In conclusion, we prove the efficacy of NFPX decoction in the treatment of ARDS. Furthermore, integrating network pharmacology, transcriptome, and artificial intelligence analysis contributes to illustrating the molecular mechanism of NFPX decoction on ARDS.

## Introduction

Acute lung injury/acute respiratory distress syndrome (ALI/ARDS) is a devastating clinical syndrome characterized by increased non-fluid extravascular pulmonary water, decreased pulmonary compliance, and acute hypoxic respiratory failure (Thompson et al., 2017; Cao et al., 2020). The pathophysiological changes of ALI/ARDS are represented by alveolar interstitial edema, pulmonary hemorrhage, lung consolidation, and inflammatory cells infiltration. These processes are thought to be related to many target inflammatory cells and effector cytokines (Brooks et al., 2020). As a treasury of medicine in China, traditional Chinese medicine (TCM) plays an important role in attenuating inflammation and improving immune function (Zhang and Wei, 2020).

In recent years, more and more attention has been paid to the roles of TCM in ARDS treatment. One study has shown that hydroxysafflor yellow A alleviates LPS-induced ARDS in mice by blocking TLR4/NF-κB signaling pathway (Zhang et al., 2017). In a rat model, silymarin can attenuate LPS-induced lung injury by inhibiting the MAPK signaling pathway (Zhu and Sun, 2018). Chen et al. have found that honokiol could protect the pulmonary microvascular endothelial barrier from damage by LPS in ARDS models by promoting the SIRT3/AMPK signaling pathway and suppressing Ang-2 expression (Chen et al., 2018). It was also reported that celastrol might reduce ARDS-related lung injury caused by LPS in rats by inactivating NF-κB signaling pathways (Wei and Wang, 2017). Although there are a lot of achievements achieved from the studies of TCM in ARDS, the detailed molecular mechanisms of TCM are rarely known due to the complexity and diversity of TCM ingredients and the synergistic or antagonistic effects among the ingredients. Different from the pattern of “one target, one drug” in modern medicine, TCM theory emphasizes a holistic view of the human body. Conventional experimental pharmacological techniques may not be applicable to the research field of TCM on account of the complexity of its components, targets, and mechanisms, posing challenges for the development of TCM.

The development of transcriptomics, proteomics, and metabolomics marked the beginning of the post-genomic era, which promoted the birth of network pharmacology (Pan et al., 2020). Network pharmacology is a sophisticated tool system that deciphers the mechanisms of complex herb formulas from the component level to gene level based on multiple large databases (Boezio et al., 2017). One of the most important characteristics of TCM is “holistic philosophy,” which coincides with systemic analysis of “network pharmacology.” As an advanced research method, the network pharmacology of TCM transforms the research paradigm from “one target, one drug” into the novel “network target, multi-components.” This helps assess the compatibility and cooperativity of TCM and elaborate the relationships of targets and signaling pathways in the network (Chen et al., 2016).

**FIGURE 1:**
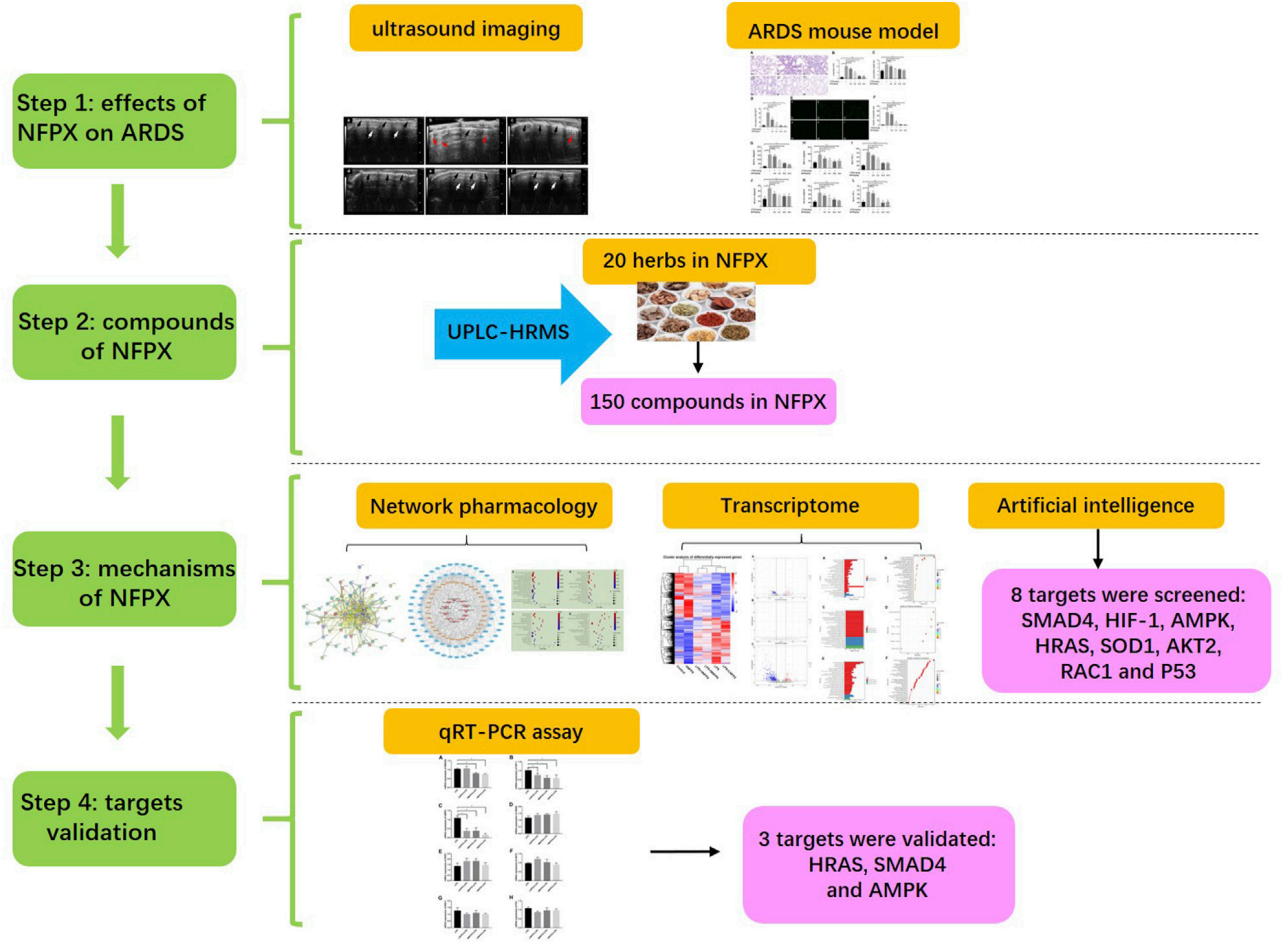
The schematic of the workflow.

Ning Fei Ping Xue (NFPX) decoction is a kind of TCM formula. It is comprised of twenty herbs: *Paeonia lactiflora* Pall. [Paeoniaceae; Paeoniae Radix Alba, 7 g], *Atractylodes macrocephala* Koidz. [Asteraceae; Atractylodis macrocephalae rhizoma, 10 g], *Conioselinum anthriscoides* “Chuanxiong” [Apiaceae; Chuanxiong Rhizoma, 7 g], *Angelica sinensis* (Oliv.) Diels [Apiaceae; Angelicae Sinensis Radix, 7 g], *Poria cocos* (Schw.) Wolf [Polyporaceae; Poria, 10 g], *Carthamus tinctorius* L. [Asteraceae; Carthami Flos, 7 g], *Phellodendron chinense* C.K.Schneid. [Rutaceae; Phellodendri Chinrnsis Cortex, 10 g], *Coptis chinensis* Franch. [Ranunculaceae; Coptidis Rhizoma, 10 g], *Astragalus mongholicus* Bunge [Fabaceae; Astragali radix, 80 g], *Scutellaria baicalensis* Georgi [Lamiaceae; Scutellariae Radix, 10 g], *Phragmites australis* (Cav.) Trin. ex Steud. [Poaceae; Phragmitis Rhizoma, 10 g], *Gardenia jasminoides* J.Ellis [Rubiaceae; Gardeniae Fructus, 10 g], *Rehmannia glutinosa* (Gaertn.) DC. [Orobanchaceae; Rehmanniae Radix Praeparata, 10 g], *Prunus persica* (L.) Batsch [Rosaceae; Persicae Semen, 7 g], *Descurainia sophia* (L.) Webb ex Prantl [Brassicaceae; Descurainiae semen lepidii semen, 5 g], *Coix lacryma-jobi* var. *ma-yuen* (Rom.Caill.) Stapf [Poaceae; Coicis Semen, 10 g], *Alisma plantago-aquatica* subsp. *orientale* (Sam.) Sam. [Alismataceae; Alismatis rhizoma, 15 g], *Polyporus umbellatus* (Pers) Fr. [Polyporaceae; Polyporus, 10 g], *Neolitsea cassia* (L.) Kosterm. [Lauraceae; Cinnamomi cortex, 7 g], and Pheretima, 7 g. NFPX decoction has been found to mitigate the inflammatory response of acute and chronic respiratory diseases in clinical practice. Improved oxygen saturation, increased number of ventilator-free days, and shortened ICU and hospital lengths of stay were observed in patients with respiratory failure after the administration of NFPX decoction. However, its efficacy in ARDS and specific molecule target and mechanism still need to be investigated. In the present study, we have first investigated the effects of NFPX decoction on ameliorating lung edema and inflammatory response of ARDS mice induced by lipopolysaccharide (LPS). Additionally, we have explored the mechanisms by screening specific molecular targets using network pharmacology, transcriptome analysis, and artificial intelligence analysis to provide the theoretical basis for the clinical application of NFPX decoction on ARDS patients. The detailed schematic of the workflow in the current study is shown in Figure 1.

## Materials and Methods

### Acute Respiratory Distress Syndrome (ARDS) Murine Model

Eight-week-old male C57BL/6N mice were purchased from Vital River Animal Institute (Beijing, China) and were maintained under specific pathogen-free (SPF) conditions. The mice were randomly divided into six groups (five mice per group): Control, LPS+PBS, LPS+2.6 g/kg NFPX (LNFPX), LPS+5.2 g/kg NFPX (MNFPX), LPS+10.4 g/kg NFPX (HNFPX), and 10.4 g/kg NFPX. Doses of LPS (2 mg/kg) and NFPX (2.6, 5.2, and 10.4 g/kg) were chosen according to previous reports and our pilot studies (Lang et al., 2017). NFPX granules were kindly provided by Prof. Jianxin Chen (Beijing University of Chinese Medicine, Beijing, China), which were extracted by ethanol (Bu et al., 2020). The extraction procedures are as follows: water and ground NFPX material were placed in a glass tube (12:1); the solution was kept boiling for 1 h; then, water (8:1) was added for second water extraction step. The water supernatant was filtered and dried using a rotary evaporator under vacuum followed by freeze-drying to obtain the water extract. 55% ethanol was added to water extract in the glass tube, and the mixture was sonicated for 1 h. The ethanol extract was filtered through a 0.45 µm syringe filter; then, the extract was made into granules. Mice were anesthetized with gaseous isoflurane, followed by instillation of 40 μl LPS (*Escherichia coli* 055: B5, L8880; Solarbio, Beijing, China) into the tracheas using 22G needles to establish ARDS model or 40 μl PBS as control. For NFPX pretreatment, various doses of NFPX granules dissolved in water were administered intragastrically daily for 7 consecutive days before LPS administration. On day 8, the animals were anesthetized with gaseous isoflurane; retroorbital venous blood, BALF, and lung tissues were collected for the subsequent analysis.

### Lung Ultrasound of Mice

Lung ultrasound was performed using a high-resolution Vevo2100 Ultrasound System (Visualsonics Inc., Toronto, Canada) with an ultrahigh-frequency (40 MHz) transducer probe to obtain a maximum resolution of 30 µm and imaging depth of 10.0 mm. The hair of the anterior chest in mice was removed by depilatory cream after 24 h exposure to LPS. Lung ultrasound videos were recorded and analyzed by two expert technicians (Shanshan Zhang and Xiaoming Dong).

### Hematoxylin and Eosin (HE) and TUNEL Staining

For histological examination, the left lung lobes were perfused with 4% paraformaldehyde and embedded in paraffin. Four-micron thick slides were stained with HE and were reviewed by two skilled pathologists. To quantify the lung injury and inflammation response, a semiquantitative histology score method was adopted (Dai et al., 2018). Briefly, alveolar edema, pulmonary hemorrhage, atelectasis, and inflammatory cells infiltration were each scored on a 0–4 scale. The total score was then calculated by adding the scores of all four histological indexes. The apoptotic cells of mouse samples were detected by the TUNEL kit (Roche, Indianapolis, United States) according to the manufacturer’s instructions. Controls were set with PBS instead of the primary antibody.

### Lung Wet/Dry (W/D) Weight Ratio

The wet/Dry ratio is an indicator of pulmonary edema by calculating extravascular lung water. Lung lobes were harvested and weighted as soon as possible to get the “wet weight.” Then, lung tissues are placed in an incubator at 65°C for 48 h and re-weighed to get the “dry weight.” Lung wet/Dry ratio = wet weight divided by dry weight.

### Enzyme-Linked Immunosorbent Assay (ELISA)

Retroorbital venous blood was collected into 2 ml Eppendorf tubes. The tubes were left at room temperature until the blood had clotted. Serum was separated by centrifugation at 1000 × *g* for 15 min. Moreover, BALF was collected by intratracheally administering 1 ml of PBS. The concentrations of IL-1β, IL-6, and TNF-α in serum and BALF were determined by ELISA kits (Cusabio, Wuhan, China) according to the manufacturer’s protocols.

### Ultra-Performance Liquid Chromatography–High-Resolution Mass Spectrometry (UPLC-HRMS) Analysis

The UPLC system was performed on an Agilent 1290 LC system (Agilent Technologies Inc., Palo Alto, CA, United States) equipped with a binary pump, a thermostat-controlled column compartment, an autosampler, and a DAD detector. Waters ACQUITY UPLC CSH C18 (2.1 × 100 mm, 1.7 μm) was employed at 30°C with sample injection volume of 3 μl. The mobile phase consisted of 0.1% formic acid in water (A) and 0.1% formic acid in acetonitrile (B) using gradient program at a flow rate of 0.3 ml/min and was eluted with gradient elution program as follows: 0–5 min (5% B), 5–8 min (5–10% B), 8–18 min (10% B), 18–23 min (10–17% B), 23–26 min (17–20% B), 26–44 min (20–28%% B), 44–46 min (28–40%% B), 46–56 min (40–60% B), 56–60 min (60–95% B), 60–63 min (95% B), 63–63.1 min (95–5% B), and 63.1–65 min (5% B) protocol. The Mass Spectrometer AB Sciex TripleTOF 4600 (AB SCIEX, Foster City, CA, United States), equipped with an electrospray ionization (ESI) source, was controlled by Analyst TF 1.7.1. software (AB SCIEX, Foster City, CA, United States). The spectrometer was operated in full-scan TOF-MS at m/z 50–1700 and information-dependent acquisition (IDA)MS/MS modes, with negative and positive ionization modes. The optimized parameters of mass spectrometry as follows: Ion Source Temperature was 500°C; Curtain Gas was 35 psi; Ion Source Gas 1 and 2 were 50 psi; Ion Spray Voltage was 5000 V (positive)/4500 V (negative); Declustering Potential was 100 V (MS and MS/MS); Collision Energy was 40 eV; Collision Energy Spread was 20 eV (MS/MS); mass range was 50–1700 m/z (MS)/50–1250 m/z (MS/MS); Ion Release Delay was 30 ms; Ion Release Width was 15 ms.

Data analysis was performed by PeakView 1.2 software (AB SCIEX, Foster City, CA, United States). The phytochemical compounds were tentatively characterized based on their retention time, mass accuracy of precursor ions, MS/MS spectra, and fragmentation pathways, referring to the Natural Products HR-MS/MS Spectra Library and literature report.

### Identification of Bioactive Components and Targets in NFPX Decoction

All candidate components and targets of the twenty traditional medicinal herbs in NFPX (*Paeonia lactiflora* Pall. [Paeoniaceae; Paeoniae Radix Alba, 7 g], *Atractylodes macrocephala* Koidz. [Asteraceae; Atractylodis macrocephalae rhizoma, 10 g], *Conioselinum anthriscoides* “Chuanxiong” [Apiaceae; Chuanxiong Rhizoma, 7 g], *Angelica sinensis* (Oliv.) Diels [Apiaceae; Angelicae Sinensis Radix, 7 g], *Poria cocos* (Schw.) Wolf [Polyporaceae; Poria, 10 g], *Carthamus tinctorius* L. [Asteraceae; Carthami Flos, 7 g], *Phellodendron chinense* C.K.Schneid. [Rutaceae; Phellodendri Chinrnsis Cortex, 10 g], *Coptis chinensis* Franch. [Ranunculaceae; Coptidis Rhizoma, 10 g], *Astragalus mongholicus* Bunge [Fabaceae; Astragali radix, 80 g], *Scutellaria baicalensis* Georgi [Lamiaceae; Scutellariae Radix, 10 g], *Phragmites australis* (Cav.) Trin. ex Steud. [Poaceae; Phragmitis Rhizoma, 10 g], *Gardenia jasminoides* J.Ellis [Rubiaceae; Gardeniae Fructus, 10 g], *Rehmannia glutinosa* (Gaertn.) DC. [Orobanchaceae; Rehmanniae Radix Praeparata, 10 g], Prunus persica (L.) Batsch [Rosaceae; Persicae Semen, 7 g], *Descurainia sophia* (L.) Webb ex Prantl [Brassicaceae; Descurainiae semen lepidii semen, 5 g], *Coix lacryma-jobi* var. *ma-yuen* (Rom.Caill.) Stapf [Poaceae; Coicis Semen, 10 g], *Alisma plantago-aquatica* subsp. *orientale* (Sam.) Sam. [Alismataceae; Alismatis rhizoma, 15 g], *Polyporus umbellatus* (Pers) Fr [Polyporaceae; Polyporus, 10 g], *Neolitsea cassia* (L.) Kosterm. [Lauraceae; Cinnamomi cortex, 7 g], and Pheretima, 7 g) were retrieved from the traditional Chinese medicine systems pharmacology (TCMSP) database (http://tcmspw.com/tcmsp.php) (Ru et al., 2014) and SymMap database (https://www.symmap.org) (Wu et al., 2019). Oral bioavailability (OB) is usually an essential pharmacokinetic parameter (Xu et al., 2012). As a qualitative parameter, drug-likeness (DL) plays a role in evaluating the druggability of a component (Tao et al., 2013). In the current study, we set up the compounds in NFPX with OB ≥30% and DL index ≥0.18 as bioactive ingredients, as shown in previous reports (Guo et al., 2019; Yu et al., 2020).

### Collection of Gene Symbols for ARDS and Construction of Protein–Protein Interaction (PPI) Networks

Underlying gene symbols of ARDS were obtained from two databases, namely, GeneCards database (https://www.genecards.org/) and OMIM database (http://www.omim.org/). Then, the protein targets of NFPX were mapped with ARDS using the comparative toxicogenomics database (CTD) (http://ctdbase.org/) (Davis et al., 2021). The obtained intersection genes were uploaded onto STRING 11.0 (http://string-db.org/) (Szklarczyk et al., 2019) to obtain the protein–protein interactions (PPI) network of NFPX treatment in ARDS.

### Construction of Networks and Analysis of Target Pathways

To further characterize the molecular mechanism of NFPX on ARDS, the herbs-compounds-targets network was established using Cytoscape 3.7.2 software (Bethesda, MD, United States). The potential pathways were identified by Gene Ontology (GO) enrichment analysis and the Kyoto Encyclopedia of Genes and Genomes (KEGG) pathway analysis.

### RNA-seq and Pathway Enrichment Analysis

Lung tissue samples were sent to the Beijing Genomics Institute (BGI, Shenzhen, China) for RNA extraction, cDNA library construction, qualification, further RNA-seq detection by Illumina HiSeqTM sequencing platform, and final bioinformatic analysis. Total RNA was extracted from the tissues using Trizol (Invitrogen, Carlsbad, CA, United States) according to manual instructions. Subsequently, total RNA was qualified and quantified using a NanoDrop and Agilent 2100 bioanalyzer (Thermo Fisher Scientific, MA, United States). Oligo(dT)-attached magnetic beads were used to purify mRNA. Purified mRNA was fragmented into small pieces with fragment buffer at an appropriate temperature. Then, first-strand cDNA was generated using random hexamer-primed reverse transcription, followed by second-strand cDNA synthesis. Afterward, A-Tailing Mix and RNA Index Adapters were added by incubating to end repair. The cDNA fragments obtained from the previous step were amplified by PCR, and products were purified by Ampure XP Beads and then dissolved in EB solution. The product was validated on the Agilent Technologies 2100 bioanalyzer for quality control. The double-stranded PCR products from the previous step were heated, denatured, and circularized by the splint oligo sequence to get the final library. The single-strand circle DNA (ssCir DNA) was formatted as the final library. The final library was amplified with phi29 to make DNA nanoball (DNB), which had more than 300 copies of one molecular, DNBs were loaded into the patterned nanoarray, and single-end 50 bases reads were generated on the BGIseq500 platform (BGI-Shenzhen, China). The quantitative analysis for DEGs was performed based on the GO functional and KEGG pathway analysis. log2(Fold Change) ≥ 1 and FDR ≤ 0.05 were used as the threshold for significant DEGs (Li et al., 2019; Cao et al., 2020).

### Specific Gene Module–Based Target Identification

Gene module pair–based target identification (GMPTI) approach was utilized to predict novel compound–target interactions based on a drug-induced gene expression profile (http://www.bcxnfz.top/TMP/). GMPTI considers experiments with gene expression profiles from a collection of samples belonging to two classes, for example, drug-treated vs. control. The genes can be ordered in a ranked list L, according to their differential expression between the classes. Given the defined gene module pair (GMP) for each target, the goal of GMPTI is to compare L to each target-specific gene module pair using a similarity metric slightly adjusted from that used in Gene Set Enrichment Analysis (Subramanian et al., 2005). We defined the raw similarity score as follows:

$$TCS_{L}^{t}=ES_{L}^{up}-ES_{L}^{down},$$

where $ES_{L}^{up}$ is the enrichment of tup for L and $ES_{L}^{down}$ is the enrichment of tdown for L. $TCS_{L}^{t}$ denotes the Total Correlation Score of the GMP (tup, tdown) of one target, with respect to the signatures L. TCS ranges between −2 and 2. It measures the degree of similarity between query L and target-induced gene expression profiles.

We assess the significance of an actual TCS value by comparing it with the set of scores TCSNULL computed with random permutations of both top and bottom gene modules for each target. A nominal *p* value for the TCSi of target i is estimated using the portion of the TCSNULL distribution above the actual TCSi, as follows:

$$P=\frac{N\left(abs\left(TCS_{NULL}\right)\geq abs\left(TCS_{i}\right)\right)}{1000},$$

where $abs\left(TCS_{NULL}\right)$ is the absolute values of all correlation scores for random GMPs with respect to a query gene list L. $abs\left(TCS_{i}\right)$ is the absolute value of the similarity score of target i with respect to L.

### Structural Docking of NFPX Ingredients and Potential Targets

To test interactions of NFPX ingredients and the eight potential targets, the target-structure-based docking method was utilized. Among the eight targets, we collected the known three-dimensional structures for the five targets, AMPK (PDB ID: 4cfe), HRAS (PDB ID: 6mqt), SOD1 (PDB ID: 5o40), AKT2 (PDB ID: 3d0e), and RAC1 (PDB ID: 3th5) from the PDB database (https://www.rcsb.org/). The protein structures of the other three targets, SMAD4, P53, and HIF-1, were collected from the AlphaFold Protein Structure Database (https://alphafold.ebi.ac.uk/), which includes the highly accurate protein structures predicted using AlphaFold v2.0. Then, these targets were docked by the NFPX ingredients with a three-dimensional structure on the representative conformations using the SYBYL−Surflex docking in standard precision mode.

### qRT-PCR

Total RNA was extracted from lung tissues using the RNA extraction kit (Qiagen, Hilden, Germany). qRT-PCR was performed utilizing the qRT-PCR kit (Thermo Fisher, United States) in the ABI StepOnePlus PCR system according to the manufacturer’s protocol. The ACTB mRNA expression level was employed as an internal control. The primers were designed as follows: SMAD4, forward, 5′-GTC​ATC​CTG​CTC​ACC​AGA​TGT​C-3′ and reverse, 5′-TGC​TCA​GAC​AGG​CAT​CGT​TAC-3′; HIF-1, forward, 5′-AGC​AAG​ATC​TCG​GCG​AAG​C-3′ and reverse, 5′-ACC​ACC​GGC​ATC​CAG​AAG​T-3′; MAPK, forward, 5′-ACA​GGC​AGC​GGA​GAC​ACC​TA-3′ and reverse, 5′-GGG​GAG​GAT​GAT​CGA​GAC​AC-3′; HRAS, forward, 5′-ATC​CAG​CTG​ATC​CAG​AAC​CAC-3′ and reverse, 5′-TCC​CGC​ATG​GCA​CTA​TAC​TC-3′; SOD1, forward, 5′-CAG​AAG​GCA​AGC​GGT​GAA​C-3′ and reverse, 5′-GAG​GTC​CTG​CAC​TGG​TAC​AGC-3′; AKT2, forward, 5′-TGC​TGC​CGC​CAG​TTC​ATA-3′ and reverse, 5′-GCA​GGA​GGC​TCC​TCG​GAT​AC-3′; RAC1, forward, 5′-CAG​ATG​CAG​GCC​ATC​AAG​TG-3′ and reverse, 5′-GTC​AAA​GAC​GGT​GGG​GAT​GT-3′; P53, forward, 5′-CTC​CCT​CTG​AGC​CAG​GAG​AC-3′ and reverse, 5′-GAC​ACT​CGG​AGG​GCT​TCA​CT-3′; ACTB, forward, 5′-TTC​ATG​GAT​GCC​ACA​GGA​TT-3′ and reverse, 5′-TGA​CGG​CCA​GGT​CAT​CAC​TA-3′. The qRT-PCR results were analyzed and expressed as the relative mRNA expression of the CT (threshold cycle) value, which was then converted to fold changes.

### Statistical Analysis

Values were represented as the mean ± SD, and two-tailed *t*-test was used for two preselected groups by GraphPad Prism 7.0 (GraphPad Software Inc, CA, United States). *p* value < 0.05 was considered statistically significant.

## Results

### NFPX Attenuates the Ultrasound Imaging Lesions of ARDS

**FIGURE 2:**
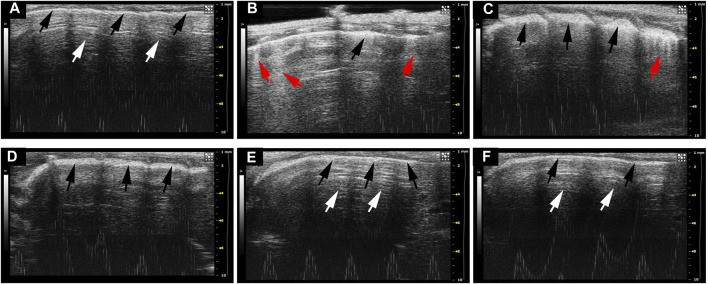
The ultrasound imaging lesions of ARDS were evaluated by lung ultrasound. **(A)** Control group: **(B)** LPS+PBS group; **(C)** LPS+2.6 g/kg NFPX group; **(D)** LPS+5.2 g/kg NFPX group; **(E)** LPS+10.4 g/kg NFPX group; **(F)** 10.4 g/kg NFPX group. White arrow: A-lines; red arrow: B-lines; black arrow: pleural line.

The typical ultrasonographic artifacts of normal lung tissues are characteristic of lung sliding with horizontal, parallel lines below the pleural line, referred to as A-lines. In contrast, lung ultrasonograms of ARDS usually show B-lines and pleural thickening and ground-glass areas (Picano et al., 2006). B-lines are defined as comet tail-like hyperechoic reverberation artifacts arising from and perpendicular to the pleural line, which is representative of thickened interlobular septa. To elucidate the imaging characteristics of different disposing groups, we performed lung ultrasound after 24 h treatment of LPS. As shown in Figure 2A, lung tissues in healthy mice showed A-lines (white arrow) and uniformly continuous pleural line (black arrow). In contrast, multiple well-defined B-lines and thickened pleural and ground-glass areas can be seen in the LPS-induced mouse model (Figure 2B). As anticipated, NFPX lightened the ultrasound abnormalities caused by LPS. It can be observed that fewer B-lines and ground-glass areas exist in LPS+2.6 g/kg NFPX or LPS+5.2 g/kg NFPX group (Figures 2C,D) than those in the LPS treatment group. What is more, a high concentration of NFPX treatment with or without LPS appears the same as that in the ultrasound images of normal mice (Figures 2E,F). These data reasonably suggested that NFPX may relieve the alveolar interstitial edema and thickened interlobular septa.

### NFPX Mitigates LPS-Induced ARDS by Inhibiting Cell Apoptosis and Inflammatory Reaction

**FIGURE 3:**
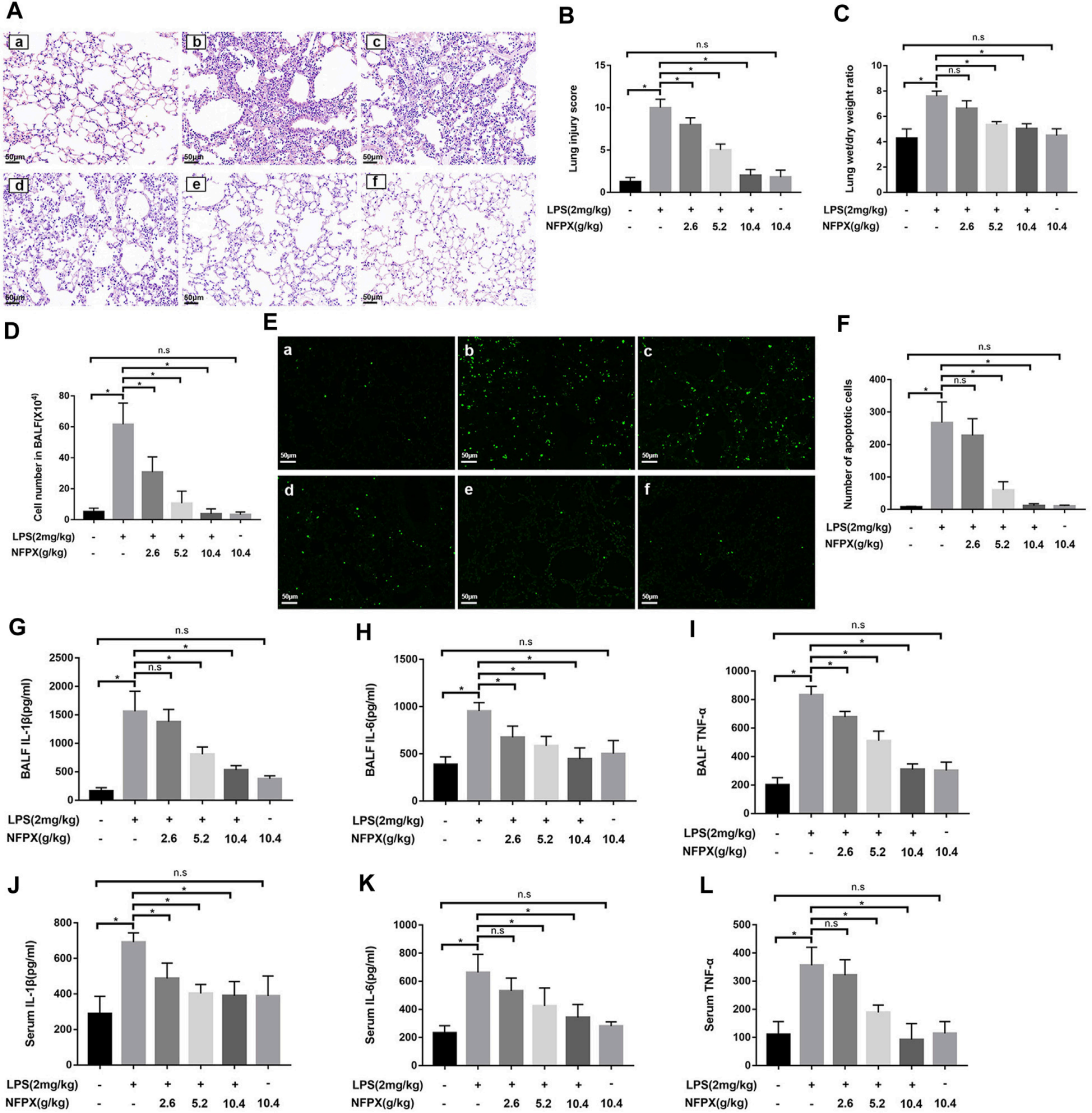
NFPX mitigates LPS-induced ARDS by inhibiting cell apoptosis and inflammatory reaction. **(A**,**B)** The lung histopathology analysis was examined by HE staining and scored by two independent pathologists. Scale bar, 50 μm. **(C)** Lung wet/dry ratios were calculated by weighting the initial weight and the dry weight. **(D)** Cell numbers in BALF were observed by cell counter. **(E**,**F)** Cell apoptosis was estimated by the TUNEL staining assay. Scale bar, 50 μm. **(G**–**I)** The levels of IL-1β, IL-6, and TNF-α in BALF were detected by the ELISA assay. **(J**–**L)** The levels of IL-1β, IL-6, and TNF-α in serum were detected by the ELISA assay. (a) Control group: (b) LPS+PBS group; (c) LPS+2.6 g/kg NFPX group; (d) LPS+5.2 g/kg NFPX group; (e) LPS+10.4 g/kg NFPX group; (f) 10.4 g/kg NFPX group. *p < 0.05.

The previous data provide intuitive evidence for NFPX exerting protective effects against ARDS. We further validated the protective effects of NFPX during experimental ARDS. HE staining was performed to assess the pathological changes of the lung. As shown in Figures 3A,B, alveolar edema, pulmonary hemorrhage, atelectasis, and inflammatory cells infiltration were the most severe in the LPS group and had the highest lung injury score correspondingly. NFPX treatment effectively alleviated these LPS-induced pathological changes in a dose-dependent manner. Compared to naïve mice, NFPX alone treatment did not exhibit significant pathological changes in tissues. Besides, several indicators associated with lung microvascular permeability and extravascular lung water were quantified, including lung wet/dry weight ratio and cell number in bronchoalveolar lavage fluid (BALF). As expected, administration with NFPX prominently reduced the lung wet/dry weight ratio and cell number in BALF induced by LPS in a dose-dependent manner (*p* < 0.05, Figures 3C,D). These results sufficiently supported that NFPX remarkably abrogated LPS-induced pathological changes without exerting side effects.

Next, we further explore how the NFPX plays a role in deterring the development of ARDS induced by LPS. Cell apoptosis and inflammation reaction are the core pathophysiologic mechanisms of ARDS. The proinflammatory cytokines, IL-1β, IL-6, and TNF-α, contribute to the infiltration of inflammatory cells during ARDS development. In accordance with the above data, NFPX also hampers the cell apoptosis and the level of cytokines in the lung during ARDS in a concentration-dependent manner (Figures 3E–I). Considering the characterization of ARDS as the systemic inflammatory reaction, the levels of three cytokines in peripheral serum were also examined. Results have shown that NFPX reduced the secretion of cytokines in serum, although the levels of IL-1β, IL-6, and TNF-α are lower than those in BALF (Figures 3J–L). These findings indicate that NFPX mitigates LPS-induced ARDS by inhibiting cell apoptosis and inflammatory reaction.

### Identification of the Major Chemical Compounds in NFPX

To achieve good resolution, selectivity and peak shape within a short analysis time, various mobile phase systems, and linear gradients were investigated. Finally, aqueous acetonitrile with 0.1% formic acid on the optimized gradient was selected as the mobile phase. The MS parameters were optimized by adjusting the ion intensity and appropriate ionization, and the optimal parameters were finally selected.

**FIGURE 4:**
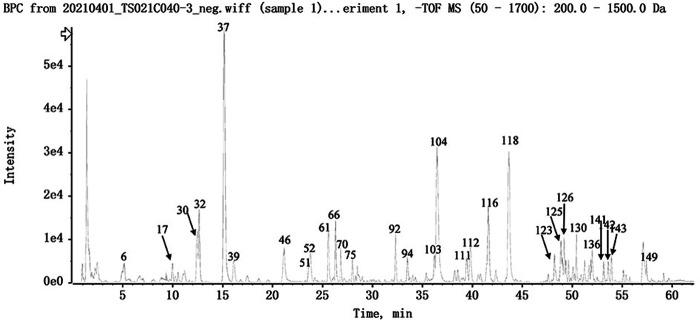
The base peak intensity chromatogram of NFPX by UPLC-HRMS in negative ion mode.

**FIGURE 5:**
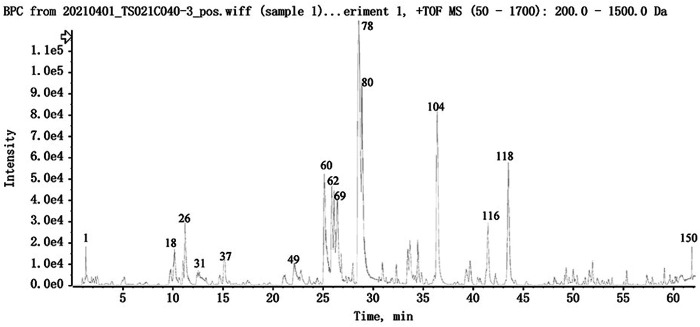
The base peak intensity chromatogram of NFPX by UPLC-HRMS in positive ion mode.

**FIGURE 6:**
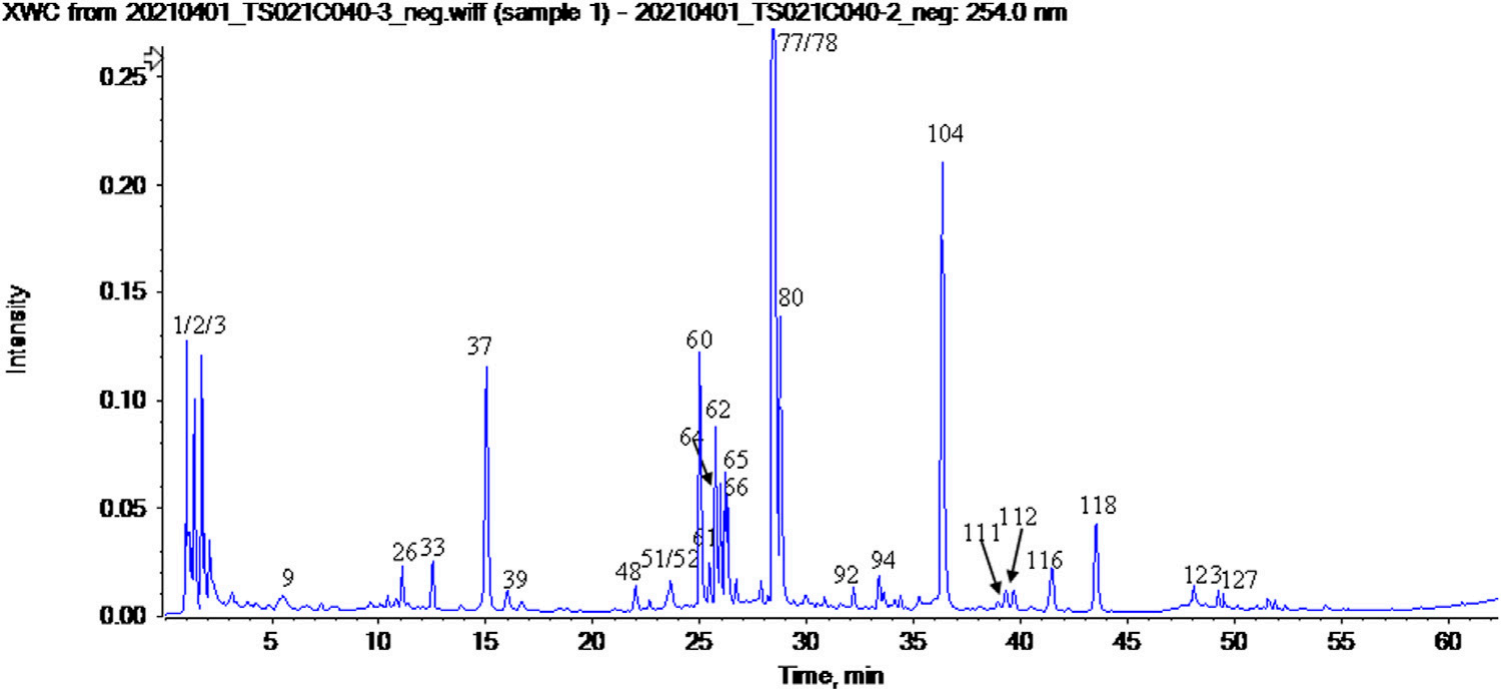
The UV chromatogram of NFPX in 254 nm.

The UPLC-HRMS method in both positive and negative ion modes was employed to characterize the major constituents in NFPX rapidly. A total of 150 compounds in NFPX were unambiguously or tentatively characterized by comparing their retention times and MS data with the Natural Products HR-MS/MS Spectral Library database or with data reported in the literature. The detailed compound information was summarized in Table 1 and the relevant chromatograms are shown in Figures 4–6, and the detailed structural formula of 150 compounds in NFPX is summarized in Supplementary Table S1.

**TABLE 1:** Identification of the major chemical compounds in NFPX.

No	RT(min)	Adduct ions	Measured m/z	Respected m/z	ppm	Formula	M.W.	Identification	Source
1	1.86	[M+H]^+^	268.1026	268.104	−5.3	C_10_H_13_N_5_O_4_	267.10	Adenosine	DL
2	2.02	[M+FA−H]^−^	407.1204	407.1195	2.2	C_15_H_22_O_10_	362.12	Catalpol	SD
3	2.19	[M+H]^+^	284.098	284.0989	−3.3	C_10_H_13_N_5_O_5_	283.09	Guanosine	DL
4	3.16	[M+H]^+^	328.1384	328.1391	−2.1	C_15_H_21_NO_7_	327.13	Fructose-phenylalanine	DL
5	3.21	[M+FA−H]^−^	731.2226	731.2251	−3.5	C_27_H_42_O_20_	686.23	Rhmannioside D	SD
6	5.11	[M−H]^−^	359.1	359.0984	4.5	C_15_H_20_O_10_	360.11	Erigeside C	HH
7	5.13	[M+Na]^+^	498.1568	498.1582	−2.8	C_20_H_29_NO_12_	475.17	O-β-D-Gentiobiosyl-D-(-)-mandelamide	TR
8	5.49	[M+H]^+^	205.0961	205.0972	−5.1	C_11_H_12_N_2_O_2_	204.09	L-Tryptophan	DL/TR
9	5.59	[M−H]^−^	391.1229	391.1246	−4.3	C_16_H_24_O_11_	392.13	Shanzhiside	SZ
10	6.72	[M+FA−H]^−^	449.1292	449.1301	−1.9	C_17_H_24_O_11_	404.13	Feretoside	SZ
11	7.06	[M+FA−H]^−^	407.1561	407.1559	0.5	C_16_H_26_O_9_	362.16	5-Deoxylamiol	SD
12	7.33	[M]^+^	314.1742	314.1751	−2.8	C_19_H_24_NO_3_	314.18	Magnocurarine	HB
13	8.11	[M+FA−H]^−^	449.13	449.1301	−0.1	C_17_H_24_O_11_	404.13	Gardenoside	SZ
14	8.48	[M+H]^+^	384.1151	384.115	0.3	C_14_H_17_N_5_O_8_	383.11	Succinyladenosine	DL
15	9.28	[M−H]^−^	475.1467	475.1457	2.1	C_20_H_28_O_13_	476.15	L-(+)-mandelic acid-O-β-D-Gentiobioside	TR
16	9.71	[M+FA−H]^−^	449.1305	449.1301	1	C_17_H_24_O_11_	404.13	Deacetyl asperulosidic acid methyl ester	SZ
17	9.98	[M−H]^−^	345.1555	345.1555	−1.1	C_16_H_26_O_8_	346.16	Jasminoside B	SZ
18	10.12	[M]^+^	342.1695	342.17	−1.4	C_20_H_24_NO_4_	342.17	Phellodendrine	HB
19	10.27	[M−H]^−^	475.1443	475.1457	−3	C_20_H_28_O_13_	476.15	D-(+)-mandelic acid-O-β-D-Gentiobioside	TR
20	10.35	[M+H]^+^	506.1994	506.2021	−5.3	C_25_H_31_NO_10_	505.19	L-Phenylalaninosecologanin B	HQs
21	10.4	[M+FA−H]^−^	493.2272	493.2291	−3.8	C_21_H_36_O_10_	448.23	Atractyloside A	BZ
22	10.53	[M+FA−H]^−^	391.1616	391.161	1.6	C_16_H_26_O_8_	346.16	Jasminoside D	SZ
23	10.94	[M−H]^−^	353.0865	353.0878	−3.7	C_16_H_18_O_9_	354.10	Neochlorogenic acid	SZ/HB/HH/DG/SZ
24	10.99	[M+H]^+^	448.1958	448.1966	−1.8	C_23_H_29_NO_8_	447.19	N-Methylhigenamine 7-glucopyranoside	HB
25	10.61	[M]^+^	344.1843	344.1856	−3.9	C_20_H_26_NO_4_	344.19	Tembetarine	HB
26	11.22	[M]^+^	342.1673	342.17	−7.8	C_20_H_24_NO_4_	342.17	Magnoflorine	HB/HL
27	11.24	[M+FA−H]^−^	477.1601	477.1614	−2.7	C_19_H_28_O_11_	432.16	8-O-Acetylmussaenoside	SZ
28	11.68	[M−H]^−^	431.155	431.1559	−2.1	C_19_H_28_O_11_	432.16	Cuchiloside	RG
29	12.16	[M−H]^−^	787.1941	787.1938	−0.1	C_33_H_40_O_22_	788.20	Quercetin3-O-β-D-glucopyranosyl-7-O-β-gentiobioside	TLZ
30	12.44	[M+FA−H]^−^	502.1558	502.1566	−1.6	C_20_H_27_NO_11_	457.16	L-Amygdalin	TR
31	12.52	[M]^+^	314.1735	314.1751	−5	C_19_H_24_NO_3_	314.18	Oblongine	HB
32	12.62	[M+FA−H]^−^	502.1582	502.1566	0.5	C_20_H_27_NO_11_	457.16	D-Amygdalin	TR
33	12.64	[M+FA−H]^−^	595.1871	595.188	−1.5	C_23_H_34_O_15_	550.19	Genipin 1-gentiobioside	SZ/SD
34	13.92	[M+H]^+^	308.091	308.0917	−2.4	C_18_H_13_NO_4_	307.08	Lycoranine B	HL
35	14.7	[M]^+^	356.1836	356.1856	−5.7	C_21_H_26_NO_4_	356.19	Menisperine	HB
36	14.96	[M−H]^−^	353.0871	353.0878	−2	C_16_H_18_O_9_	354.10	Chlorogenic acid	SZ/HB/HH/DG/SZ
37	15.15	[M+FA−H]^−^	433.1366	433.1352	3.3	C_17_H_24_O_10_	388.14	Geniposide	SZ/SD
38	15.6	[M+H]^+^	504.2224	504.2228	−0.8	C_26_H_33_NO_9_	503.22	(13aS)-5,8,13,13a-Tetrahydro-3,9,10-trimethoxy-6H-dibenzo[a,g]quinolizin-2-yl β-D-glucopyranoside	HL
39	16.14	[M−H]^−^	367.103	367.1035	5.3	C_17_H_20_O_9_	368.11	5-O-Feruloylquinic acid	HB
40	16.81	[M−H]^−^	353.0864	353.0878	−4	C_16_H_18_O_9_	354.10	Cryptochlorogenic acid	SZ/HB/HH/DG/SZ
41	17.45	[M+H]^+^	377.1452	377.1456	−1	C_17_H_20_N_4_O_6_	376.14	Vitamin B2	LG
42	17.47	[M+FA−H]^−^	525.1624	525.1614	2	C_23_H_28_O_11_	480.16	Albiflorin	BS
43	17.63	[M]^+^	356.1849	356.1856	−2.1	C_21_H_26_NO_4_	356.19	5,6,6a,7-Tetrahydro-11-hydroxy-1,2,10-trimethoxy-6,6-dimethyl-4H-dibenzo[de,g]quinolinium	HB
44	18.65	[M+FA−H]^−^	375.1649	375.1661	−3.1	C_16_H_26_O_7_	330.17	Epijasminoside A	SZ
45	19.53	[M+FA−H]^−^	375.1656	375.1661	−1.2	C_16_H_26_O_7_	330.17	Picrocrocin	SZ
46	21.14	[M+FA−H]^−^	525.1647	525.1614	6.4	C_23_H_28_O_11_	480.16	Paeoniflorin	BS
47	21.39	[M+H]^+^	356.1843	356.1856	−3.7	C_21_H_25_NO_4_	355.18	Tetrahydropalmatine	HL/HB
48	22.14	[M+H]^+^	352.1059	352.1179	−5.5	C_20_H_17_NO_5_	351.11	8-Oxoepiberberine	HL/HB
49	22.2	[M]^+^	322.1057	322.1074	−5.2	C_19_H_16_NO_4_	322.11	Groenlandicine	HL
50	22.81	[M]^+^	324.1227	324.123	−1	C_19_H_18_NO_4_	324.12	Demethyleneberberine	HL/HB
51	23.63	[M−H]^−^	611.1627	611.1618	1.5	C_27_H_32_O_16_	612.17	Hydroxysafflor Yellow A	HH
52	23.78	[M−H]^−^	367.1027	367.1035	−2.1	C_17_H_20_O_9_	368.11	3-O-Feruloylquinic acid	HB
53	24.05	[M+H]^+^	260.1272	260.1281	−3.5	C_15_H_17_NO_3_	259.12	Platydesmine	HB
54	24.21	[M−H]^−^	543.1181	543.1144	5.5	C_26_H_24_O_13_	544.12	Hyemaloside B	BS
55	24.23	[M]^+^	356.1842	356.1856	−4	C_21_H_26_NO_4_	356.19	N-Methylcorydine	HL/HB
56	24.32	[M−H]^−^	771.2008	771.1989	2.4	C_33_H_40_O_21_	772.21	Quercetin 3-O-glucosyl-rutinoside	TLZ
57	24.43	[M]^+^	354.1687	354.17	−3.6	C_21_H_24_NO_4_	354.17	N-Methylcanadine	HL/HB
58	24.48	[M−H]^−^	625.141	625.141	0	C_27_H_30_O_17_	626.15	Quercetin7-O-β-gentiobioside	TLZ
59	24.57	[M−H]^−^	785.2522	785.251	1.6	C_35_H_46_O_20_	786.26	Purpureaside C	SD
60	25.1	[M]^+^	320.0911	320.0917	−2	C_19_H_14_NO_4_	320.09	Coptisine	HL
61	25.58	[M−H]^−^	367.1027	367.1035	−2.1	C_17_H_20_O_9_	368.11	4-O-Feruloylquinic acid	HB
62	25.86	[M]^+^	336.1212	336.123	−5.5	C_20_H_18_NO_4_	336.12	Epiberberine	HL/HB
63	25.99	[M−H]^−^	385.1152	385.114	3.1	C_17_H_22_O_10_	386.12	2-Hydroxyethyl, 6-[(2E)-3-(3,4-dihydroxyphenyl)-2-propenoate]-β-D-glucopyranoside	BZ
64	26.03	[M−H]^−^	193.0521	193.0506	7.6	C_10_H_10_O_4_	194.06	Ferulic Acid	CX/DG/LG
65	26.09	[M]^+^	338.1373	338.1387	−4.1	C_20_H_20_NO_4_	338.14	Columbamine	HL/HB
66	26.3	[M+FA−H]^−^	491.1208	491.1195	2.6	C_22_H_22_O_10_	446.12	Calycosin-7-glucoside	HQ
67	26.37	[M−H]^−^	799.2677	799.2666	1.4	C_36_H_48_O_20_	800.27	Jionoside A1	SD
68	26.4	[M−H]^−^	547.1475	547.1457	3.3	C_26_H_28_O_13_	548.15	Chrysin-6-C-hexoside -8-C- pentoside	HQs
69	26.42	[M]^+^	338.1375	338.1387	−3.5	C_20_H_20_NO_4_	338.14	Jateorhizine	HL/HB
70	26.82	[M−H]^−^	547.1451	547.1457	−1.1	C_26_H_28_O_13_	548.15	Chrysin-6-C-glucoside-8-C-arabinoside	HQs
71	27.17	[M−H]^−^	547.1492	547.1457	6.4	C_26_H_28_O_13_	548.15	Chrysin-6-C-hexoside -8-C- pentoside	HQs
72	27.47	[M+H]^+^	352.1163	352.1179	−4.7	C_20_H_17_NO_5_	351.11	Oxoberberine	HL/HB
73	27.73	[M−H]^−^	547.1472	547.1457	2.7	C_26_H_28_O_13_	548.15	Chrysin-6-C-pentoside-8-C-hexoside	HQs
74	27.92	[M−H]^−^	631.1714	631.1668	7.2	C_30_H_32_O_15_	632.17	Galloylpaeoniflorin	BS
75	27.99	[M−H]^−^	547.148	547.1457	4.2	C_26_H_28_O_13_	548.15	Chrysin-6-C-arabinoside-8-C-glucoside	HQs
76	28.31	[M−H]^−^	609.1492	609.1461	5.1	C_27_H_30_O_16_	610.15	Rutin	HH
77	28.44	[M−H]^−^	623.198	623.1981	−0.2	C_29_H_36_O_15_	624.21	Aceteoside	SD
78	28.52	[M]^+^	336.1212	336.123	−1	C_20_H_18_NO_4_	336.12	Berberine	HL/HB
79	28.67	[M−H]^−^	547.147	547.1457	2.3	C_26_H_28_O_13_	548.15	Chrysin-6-C-pentoside-8-C-hexoside	HQs
80	28.82	[M]^+^	352.1529	352.1543	−4.1	C_21_H_22_NO_4_	352.15	Palmatine	HB
81	28.98	[M+FA−H]^−^	579.1723	579.1719	0.6	C_26_H_30_O_12_	534.17	Amurensin	HB
82	29.76	[M+FA−H]^−^	671.2206	671.2193	2	C_29_H_38_O_15_	626.22	Isomucronulatol-7,2′-di-O-glucoside	HQ
83	29.86	[M−H]^−^	623.2002	623.1981	3.3	C_29_H_36_O_15_	624.21	Isoaceteoside	SD
84	30.02	[M−H]^−^	461.0709	461.0725	−3.6	C_21_H_18_O_12_	462.08	Scutellarin	HQs
85	30.15	[M+FA−H]^−^	537.2182	537.2189	−1.3	C_22_H_36_O_12_	492.22	Jasminoside I/H/S	SZ
86	30.21	[M−H]^−^	491.1226	491.1254	−5.6	C_23_H_24_O_12_	492.13	Eupatolin	HH
87	30.03	[M+H]^+^	207.101	207.1016	−2.8	C_12_H_14_O_3_	206.09	Senkyunolide F	CX
88	30.36	[M+FA−H]^−^	507.1116	507.1144	−5.6	C_22_H_22_O_11_	462.12	Pratensein-7-O-glucoside	HQ
89	30.61	[M−H]^−^	593.1523	593.1512	1.9	C_27_H_30_O_15_	594.16	Kaempferol-3-O-rutinoside	TLZ/HH
90	30.92	[M]^+^	350.1367	350.1387	−5.7	C_21_H_20_NO_4_	350.14	Fagaronine	HB
91	31.94	[M+FA−H]^−^	507.1505	507.1508	0.2	C_23_H_26_O_10_	462.15	Lactiflorin	BS
92	32.31	[M−H]^−^	755.2388	755.2404	−2.1	C_34_H_44_O_19_	756.25	6''-O-[trans-Sinapoyl] -genipin gentiobioside	SZ
93	32.87	[M−H]^−^	725.2311	725.2298	1.7	C_33_H_42_O_18_	726.24	6''-O-[trans-Feruloyl] genipin gentiobioside	SZ
94	33.48	[M+FA−H]^−^	475.1248	475.1246	0.5	C_22_H_22_O_9_	430.13	Ononin	HQ
95	33.72	[M+FA−H]^−^	1021.3796	1021.377	2.6	C_44_H_64_O_24_	976.38	Crocin I	SZ
96	34.02	[M−H]^−^	551.2156	551.2134	4	C_27_H_36_O_12_	552.22	6′-O-trans-Sinapoyljasminoside L	SZ
97	34.3	[M−H]^−^	431.0987	431.0984	0.8	C_21_H_20_O_10_	432.11	Apigenin-7-O- β-D-glucoside	HH
98	35.35	[M−H]^−^	559.1479	559.1457	3.9	C_27_H_28_O_13_	560.15	3-O-Sinapoyl-5-O-caffeoylquinic acid	SZ
99	35.38	[M−H]^−^	551.2161	551.2134	4.9	C_27_H_36_O_12_	552.22	6′-O-trans-Sinapoyljasminoside L Isomer	SZ
100	35.38	[M−H]^−^	345.0607	345.0616	−2.6	C_17_H_14_O_8_	346.07	Viscidulin III	HQs
101	35.63	[M−H]^−^	593.1883	593.1876	1.2	C_28_H_34_O_14_	594.19	6′-O-sinapoylgeniposide	SZ
102	36.08	[M−H]^−^	475.0876	475.0882	−1.3	C_22_H_20_O_12_	476.10	5,7,2′-Trihydroxy-6-methoxy flavone-7-*O-*glucuronide	HQs
103	36.21	[M+FA−H]^−^	507.1509	507.1508	0.2	C_23_H_26_O_10_	462.15	Methylnissolin 3-O-glucoside	HQ
104	36.48	[M−H]^−^	445.0777	445.0776	0.8	C_21_H_18_O_11_	446.08	Baicalin	HQs
105	38.23	[M−H]^−^	447.094	447.0933	1.6	C_21_H_20_O_11_	448.10	Dihydrobaicalin	HQs
106	38.32	[M−H]^−^	559.1444	559.1457	−2.4	C_27_H_28_O_13_	560.15	4-O-sinapoyl-5-O-caffeoylquinic acid	SZ
107	38.53	[M−H]^−^	463.1628	463.161	3.9	C_23_H_28_O_10_	464.17	Isomucronulatol-7-O-glucoside	HQ
108	38.9	[M−H]^−^	447.0936	447.0933	0.7	C_21_H_20_O_11_	448.10	Naringenin-7-O-glucuronide	HQs
109	39.03	[M−H]^−^	659.1601	659.1618	−2.5	C_31_H_32_O_16_	660.17	3,5-Di-O-caffeoyl-4-O-(3-hydroxy-3-methyl) glutaroylquinic acid	SZ
110	39.11	[M−H]^−^	559.148	559.1457	0.7	C_27_H_28_O_13_	560.15	3-O-Sinapoyl-4-O-caffeoylquinic acid	SZ
111	39.41	[M−H]^−^	283.0622	283.0612	3.5	C_16_H_12_O_5_	284.07	Calycosin	HQ
112	39.79	[M−H]^−^	445.0788	445.0776	2.6	C_21_H_18_O_11_	446.08	Norwogonin 7-O-β-D-glucuronide	HQs
113	40.57	[M−H]^−^	475.0893	475.0882	2.3	C_22_H_20_O_12_	476.10	Diosmetin 7-O-β-D-glucuronide	SD
114	40.79	[M−H]^−^	445.0792	445.0776	3.5	C_21_H_18_O_11_	446.08	Baicalein 6-O-β-D-glucuronide	HQs
115	41.42	[M−H]^−^	429.0837	429.0827	2.3	C_21_H_18_O_10_	430.09	Chrysin7-O-β-D-glucuronide	HQs
116	41.59	[M−H]^−^	459.0926	459.0933	−1.5	C_22_H_20_O_11_	460.10	Oroxylin A 7-O-glucuronide	HQs
117	42.35	[M−H]^−^	475.088	475.0882	−0.4	C_22_H_20_O_12_	476.10	5,6,7-Trihydroxy-8-methoxyflavone-7-O-glucuronopyranoside	HQs
118	43.65	[M−H]^−^	459.0921	459.0933	−2.6	C_22_H_20_O_11_	460.10	Wogonoside	HQs
119	47.39	[M+H]^+^	947.5185	947.521	−2.6	C_47_H_78_O_19_	946.51	Astragaloside VI	HQ
120	47.63	[M−H]^−^	299.0566	299.0561	1.6	C_16_H_12_O_6_	300.06	3′,5,7-Trihydroxy-4′-methoxyflavone	SD
121	47.8	[M+H]^+^	191.1056	191.1067	−5.5	C_12_H_14_O_2_	190.10	3-N-butylphthalide	CX/DG
122	48.07	[M+FA−H]^−^	1021.3796	1021.377	2.6	C_44_H_64_O_24_	976.38	Crocin I	SZ
123	48.19	[M−H]^−^	269.0465	269.0455	3.5	C_15_H_10_O_5_	270.05	Baicalein	HQs
124	48.58	[M+H]^+^	947.5211	947.521	0.1	C_47_H_78_O_19_	946.51	Astragaloside VI isomer	HQ
125	48.87	[M−H]^−^	329.2344	329.2333	3.2	C_18_H_34_O_5_	330.24	Pinellic acid	/
126	49.18	[M+FA−H]^−^	829.4589	829.4591	−0.3	C_41_H_68_O_14_	784.46	Astragaloside IV	HQ
127	49.34	[M+FA−H]^−^	549.3416	549.3433	−8.6	C_30_H_48_O_6_	504.35	16-Oxoalisol A	ZX
128	49.62	[M+FA−H]^−^	697.2698	697.2713	−2.2	C_32_H_44_O_14_	652.27	Crocin Ⅲ	SZ
129	50.07	[M+FA−H]^−^	515.1925	515.1923	0.4	C_26_H_30_O_8_	470.19	Limonin	HH
130	50.38	[M+H]^+^	827.4476	827.4787	−3.4	C_43_H_70_O_15_	826.47	Astragaloside Ⅱ	HQ
131	50.88	[M−H]^−^	651.2668	651.2658	1.5	C_32_H_44_O_14_	652.27	Crocin Ⅲ isomer	SZ
132	51.01	[M+H]^+^	547.3624	547.3629	−1	C_32_H_50_O_7_	546.36	23-Acetyl 16-oxoalisol A	ZX
133	51.24	[M+H]^+^	827.4781	827.4787	−0.8	C_43_H_70_O_15_	826.47	Isoastragaloside Ⅱ	HQ
134	51.68	[M−H]^−^	283.0616	283.0612	1.4	C_16_H_12_O_5_	284.07	Wogonin	HQs
135	51.86	[M+H]^+^	827.4789	827.4787	0.2	C_43_H_70_O_15_	826.47	Cyclosiversioside D	HQ
136	52	[M−H]^−^	373.0908	373.0929	−5.6	C_19_H_18_O_8_	374.10	Skullcapflavone II	HQs
137	52.09	[M+H]^+^	193.1217	193.1223	−3.1	C_12_H_16_O_2_	192.12	Senkyunolide A	CX/DG
138	52.49	[M−H]^−^	283.0623	283.0612	3.9	C_16_H_12_O_5_	284.07	Oroxylin A	HQs
139	52.64	[M+H]^+^	231.1377	231.138	−1.1	C_15_H_18_O_2_	230.13	Atractylenolide III	BZ
140	52.83	[M+H]^+^	487.34	487.3418	−3.7	C_30_H_46_O_5_	486.33	Alisol C	ZX
141	53.08	[M+H]^+^	869.4859	869.4893	−3.9	C_45_H_72_O_16_	868.48	Astragaloside I	HQ
142	53.6	[M−H]^−^	311.2233	311.2228	1.7	C_18_H_32_O_4_	312.23	12,13-Dihydroxy-9Z,15Z-octadecadienoic acid	/
143	53.86	[M+H]^+^	869.4908	869.4893	3.1	C_45_H_72_O_16_	868.48	Isoastragaloside I	HQ
144	54.84	[M−H]^−^	519.333	519.3327	0.5	C_30_H_48_O_7_	520.34	Alisol P	ZX
145	55.05	[M+H]^+^	869.4896	869.4893	0.3	C_45_H_72_O_16_	868.48	Neoastragaloside I	HQ
146	55.13	[M+H]^+^	191.1065	191.1067	−0.8	C_12_H_14_O_2_	190.10	Ligustilide	CX/DG
147	55.32	[M+FA−H]^−^	573.3455	573.3433	3.8	C_32_H_48_O_6_	528.35	23-Acetyl alisol C	ZX
148	56.41	[M+H]^+^	233.1532	233.1536	−1.7	C_15_H_20_O_2_	232.15	Atractylenolide II	BZ
149	57.41	[M+FA−H]^−^	535.3641	535.364	0.1	C_30_H_50_O_5_	490.37	Alisol A	ZX
150	61.84	[M+H]^+^	515.3707	515.3731	−4.7	C_32_H_50_O_5_	514.37	23-Acetyl alisol B	ZX

### Screening of Bioactive Components and Targets in NFPX on ARDS

**FIGURE 7:**
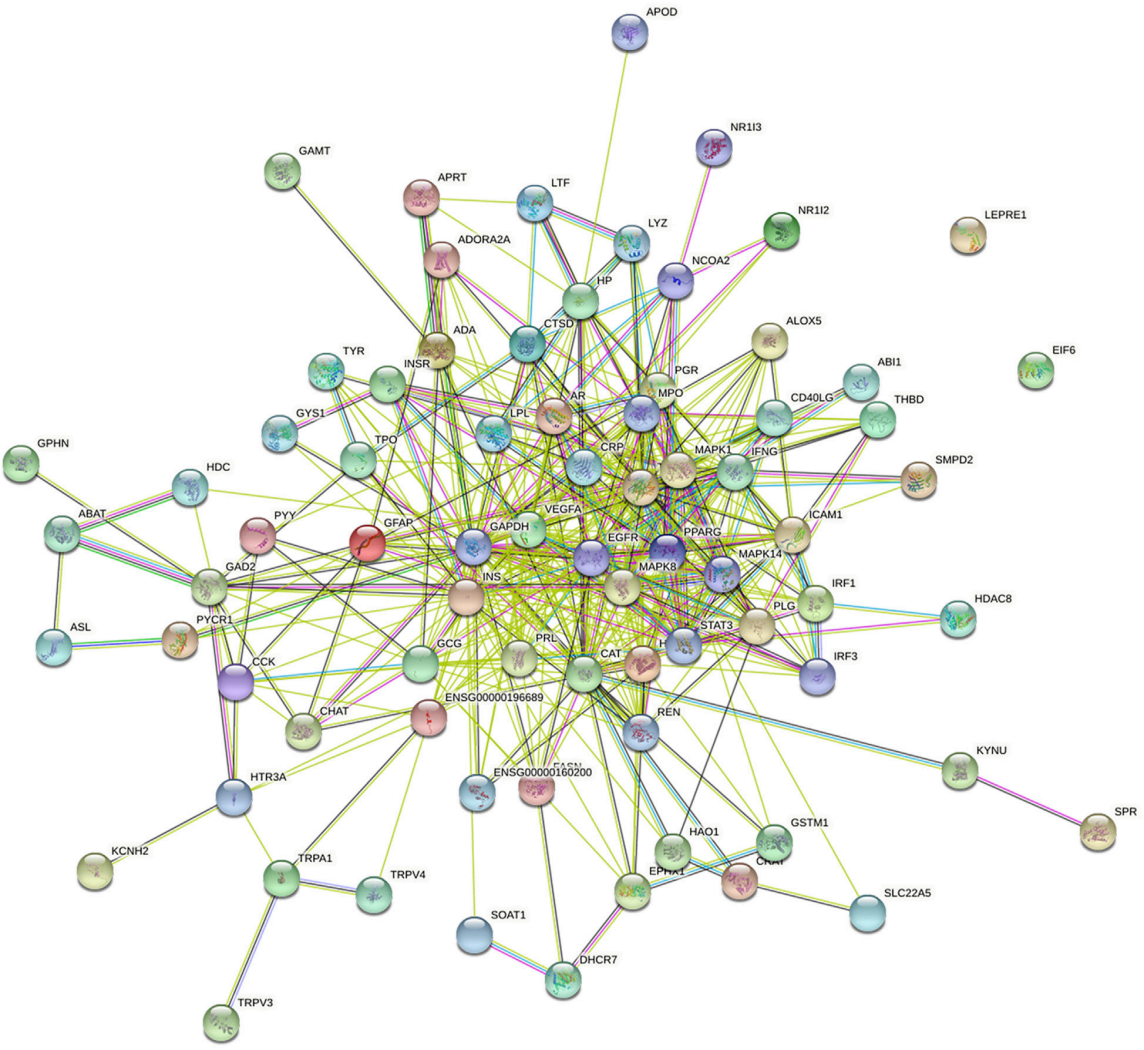
The PPI network of targets between NFPX and ARDS. Each node represents a protein and edges represent protein–protein associations.

A total of 1610 components in NFPX were obtained from the TCMSP database and SymMap database (Supplementary Table S2). OB ≥ 30% and DL index ≥0.18 served as the criteria of bioactive components. Among the 1610 components in NFPX, 821 components (51.0%) met the criterion of OB ≥ 30%, 663 components (41.2%) met the criterion of DL index ≥0.18, and 254 components (15.8%) met both criteria of OB ≥ 30% and DL index ≥0.18. Therefore, these 254 components were selected as candidate bioactive components for further analyses (Supplementary Table S3). Among the 254 candidate bioactive components, 13754 protein targets were retrieved from the TCMSP database and SymMap database (Supplementary Table S4). 3381 gene symbols for ARDS were collected from the GeneCards database and OMIM database (Supplementary Table S5). Then, gene intersections were generated by mapping the targets of NFPX with ARDS using the CTD database. Consequently, 77 targets of 37 components in NFPX associated with ARDS were obtained, and the detailed information of the 77 targets of NFPX on ARDS is shown in Table 2. PPI network was constructed to reveal the intersections of 77 target symbols using the STRING software (Figure 7).

**TABLE 2:** Targets of NFPX on ARDS were screened by network pharmacology analysis.

Herb name	Symbol	Description	Score
Atractylodis macrocephalae rhizoma	AR	Androgen receptor	39.29
Atractylodis macrocephalae rhizoma	NCOA2	Nuclear receptor coactivator 2	5.4
Carthami Flos	ADA	Adenosine deaminase	25.31
Carthami Flos	ALOX5	Arachidonate 5-lipoxygenase	35.92
Carthami Flos	APOD	Apolipoprotein D	2.28
Carthami Flos	CD40LG	CD40 Ligand	72.98
Carthami Flos	CRAT	Carnitine O-acetyltransferase	9.6
Carthami Flos	CRP	C-reactive protein	49.97
Carthami Flos	CTSD	Cathepsin D	20.47
Carthami Flos	EGFR	Epidermal growth factor receptor	42.83
Carthami Flos	EIF6	Eukaryotic translation initiation factor 6	8.88
Carthami Flos	EPHX1	Epoxide hydrolase 1	12.06
Carthami Flos	GFAP	Glial fibrillary acidic protein	28.46
Carthami Flos	GPHN	Gephyrin	21.29
Carthami Flos	GSTM1	Glutathione S-transferase Mu 1	15.39
Carthami Flos	ICAM1	Intercellular adhesion molecule 1	44.2
Carthami Flos	IFNG	Interferon gamma	61.83
Carthami Flos	INS	Insulin	60.63
Carthami Flos	INSR	Insulin receptor	19.9
Carthami Flos	IRF1	Interferon regulatory factor 1	29.12
Carthami Flos	MAPK8	Mitogen-activated protein kinase 8	20.63
Carthami Flos	NR1I2	Nuclear receptor subfamily 1 group I member 2	6.09
Carthami Flos	NR1I3	Nuclear receptor subfamily 1 group I member 3	8.89
Carthami Flos	REN	Renin	39.96
Carthami Flos	SLC22A5	Solute carrier family 22 member 5	18.11
Carthami Flos	STAT3	Signal transducer and activator of transcription 3	51.28
Carthami Flos	THBD	Thrombomodulin	53.46
Chuanxiong Rhizoma	ABI1	Abl interactor 1	4.84
Chuanxiong Rhizoma	ADORA2A	Adenosine A2a receptor	17.54
Chuanxiong Rhizoma	CCK	Cholecystokinin	15.54
Chuanxiong Rhizoma	CHAT	Choline O-acetyltransferase	38.56
Chuanxiong Rhizoma	GAD2	Glutamate decarboxylase 2	9.58
Chuanxiong Rhizoma	GAMT	Guanidinoacetate N-methyltransferase	23.69
Chuanxiong Rhizoma	GCG	Glucagon	13.57
Chuanxiong Rhizoma	HTR3A	5-Hydroxytryptamine receptor 3A	15.77
Chuanxiong Rhizoma	LPL	Lipoprotein lipase	17.68
Chuanxiong Rhizoma	MAPK14	Mitogen-activated protein kinase 14	13.1
Chuanxiong Rhizoma	PYY	Peptide YY	10.3
Cinnamomi Cortex	IRF3	Interferon regulatory factor 3	29.91
Cinnamomi Cortex	PRL	Prolactin	20.72
Cinnamomi Cortex	TRPV4	Transient Receptor Potential Cation Channel Subfamily V Member 4	26.23
Gardeniae Fructus	KCNH2	Potassium voltage-gated channel subfamily H member 2	40.48
Gardeniae Fructus	SMPD2	Sphingomyelin phosphodiesterase 2	5.43
Gardeniae Fructus	SOAT1	Sterol O-acyltransferase 1	2.07
Gardeniae Fructus	TYR	Tyrosinase	27.98
Astragali radix	FASN	Fatty acid synthase	3.77
Descurainiae semen lepidii semen	PPARG	Peroxisome proliferator-activated receptor gamma	38.7
Descurainiae semen lepidii semen	TRPA1	Transient receptor potential cation channel subfamily A member 1	15.59
Descurainiae semen lepidii semen	TRPV1	Transient receptor potential cation channel subfamily V member 1	14.15
Descurainiae semen lepidii semen	VEGFA	Vascular endothelial growth factor A	55.21
Paeoniae Radix Alba	ABAT	4-Aminobutyrate aminotransferase	4.17
Paeoniae Radix Alba	APRT	Adenine phosphoribosyltransferase	20.39
Paeoniae Radix Alba	ASL	Argininosuccinate lyase	16.65
Paeoniae Radix Alba	CAT	Catalase	31.01
Paeoniae Radix Alba	CBS	Cystathionine beta-synthase	10.92
Paeoniae Radix Alba	GAPDH	Glyceraldehyde-3-phosphate dehydrogenase	20.87
Paeoniae Radix Alba	GYS1	Glycogen synthase 1	5.42
Paeoniae Radix Alba	HAO1	Hydroxyacid oxidase 1	0.94
Paeoniae Radix Alba	HDAC8	Histone deacetylase 8	8.09
Paeoniae Radix Alba	HDC	Histidine decarboxylase	15.18
Paeoniae Radix Alba	HMOX1	Heme oxygenase 1	33.65
Paeoniae Radix Alba	HP	Haptoglobin	40.54
Paeoniae Radix Alba	KYNU	Kynureninase	9.46
Paeoniae Radix Alba	LTF	Lactotransferrin	16.34
Paeoniae Radix Alba	LYZ	Lysozyme	10.24
Paeoniae Radix Alba	MAPK1	Mitogen-activated protein kinase 1	38.57
Paeoniae Radix Alba	MMUT	Methylmalonyl-CoA mutase	16.2
Paeoniae Radix Alba	MPO	Myeloperoxidase	65.09
Paeoniae Radix Alba	PYCR1	Pyrroline-5-carboxylate reductase 1	10.26
Paeoniae Radix Alba	SPR	Sepiapterin reductase	2.82
Paeoniae Radix Alba	TPO	Thyroid peroxidase	13.14
Phellodendri Chinrnsis Cortex	TNF	Tumor necrosis factor	102.74
Phellodendri Chinrnsis Cortex	TRPV3	Transient receptor potential cation channel subfamily V member 3	7.41
Polyporus	DHCR7	7-Dehydrocholesterol reductase	17.47
Rehmanniae Radix Praeparata	P3H1	Prolyl 3-hydroxylase 1	9.44
Rehmanniae Radix Praeparata	PGR	Progesterone receptor	11.52
Rehmanniae Radix Praeparata	PLG	Plasminogen	36.75

### Herbs-Compounds-targets Network Analysis

**FIGURE 8:**
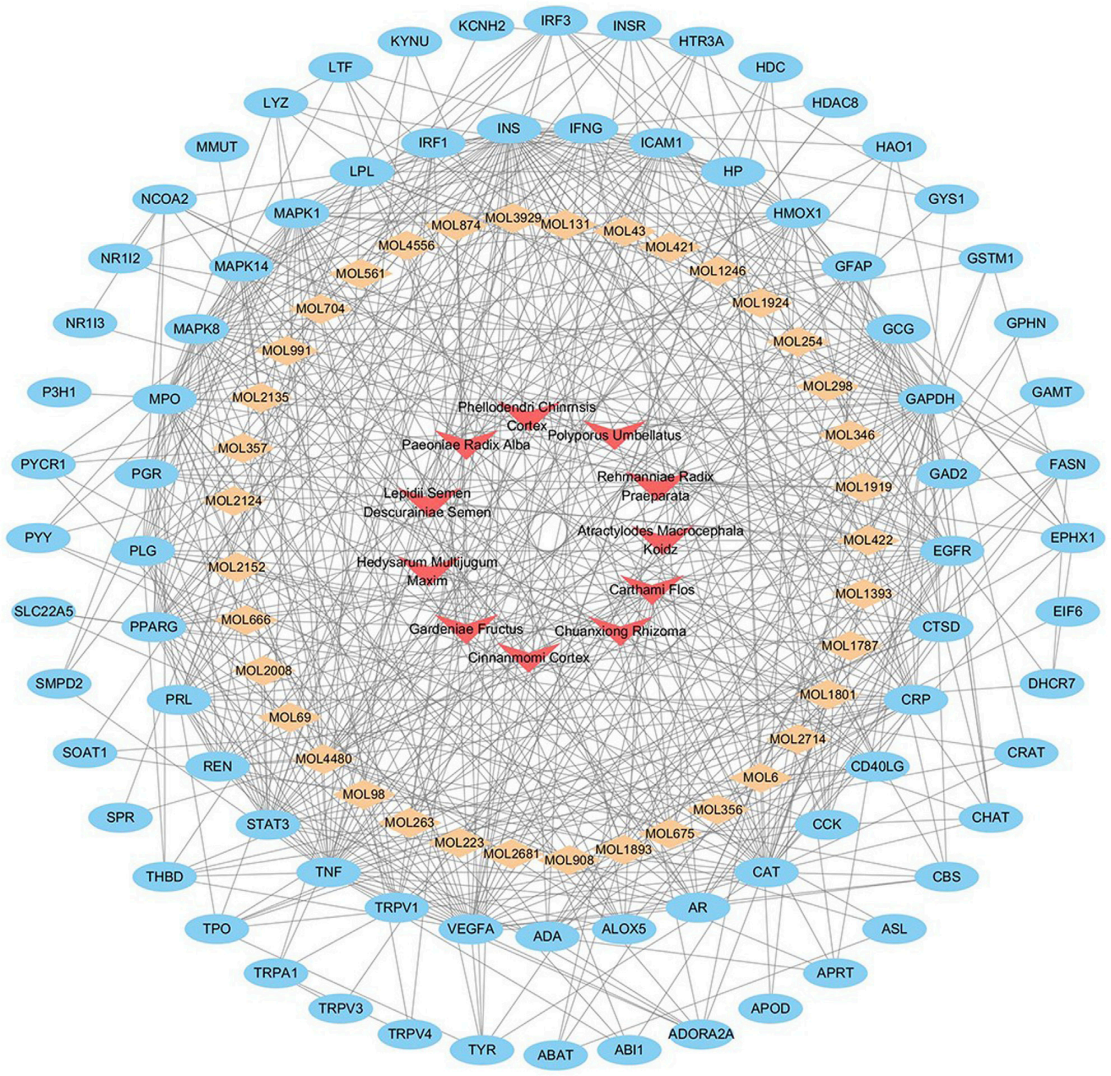
Herbs-compounds-targets network of NFPX on ARDS. The red arrow represents herbs in NFPX; the orange diamond represents the bioactive compounds of NFPX; the blue ellipse represents the target genes.

To investigate the underlying mechanisms of NFPX on ARDS, Herbs-compounds-targets network of NFPX on ARDS was constructed, which included 125 nodes and 552 edges, displaying that multiple compounds and targets are involved in the effects of NFPX treating ARDS (Figure 8). Among these bioactive components, the top five degree components associated with multiple ARDS targets include histidine decarboxylase (MOL4480, degree = 18), androgen receptor (MOL422, degree = 11), telomerase protein component 1 (MOL675, degree = 10), amine oxidase B (MOL 1801, degree = 9), nitric-oxide synthase (MOL 1893, degree = 6). In addition, the top five-degree targets related to multiple bioactive compounds include INS (degree = 47), GAPDH (degree = 40), TNF (degree = 36), VEGFA (degree = 34), CAT (degree = 33).

### Analysis of GO and KEGG Enrichment Pathway

**FIGURE 9:**
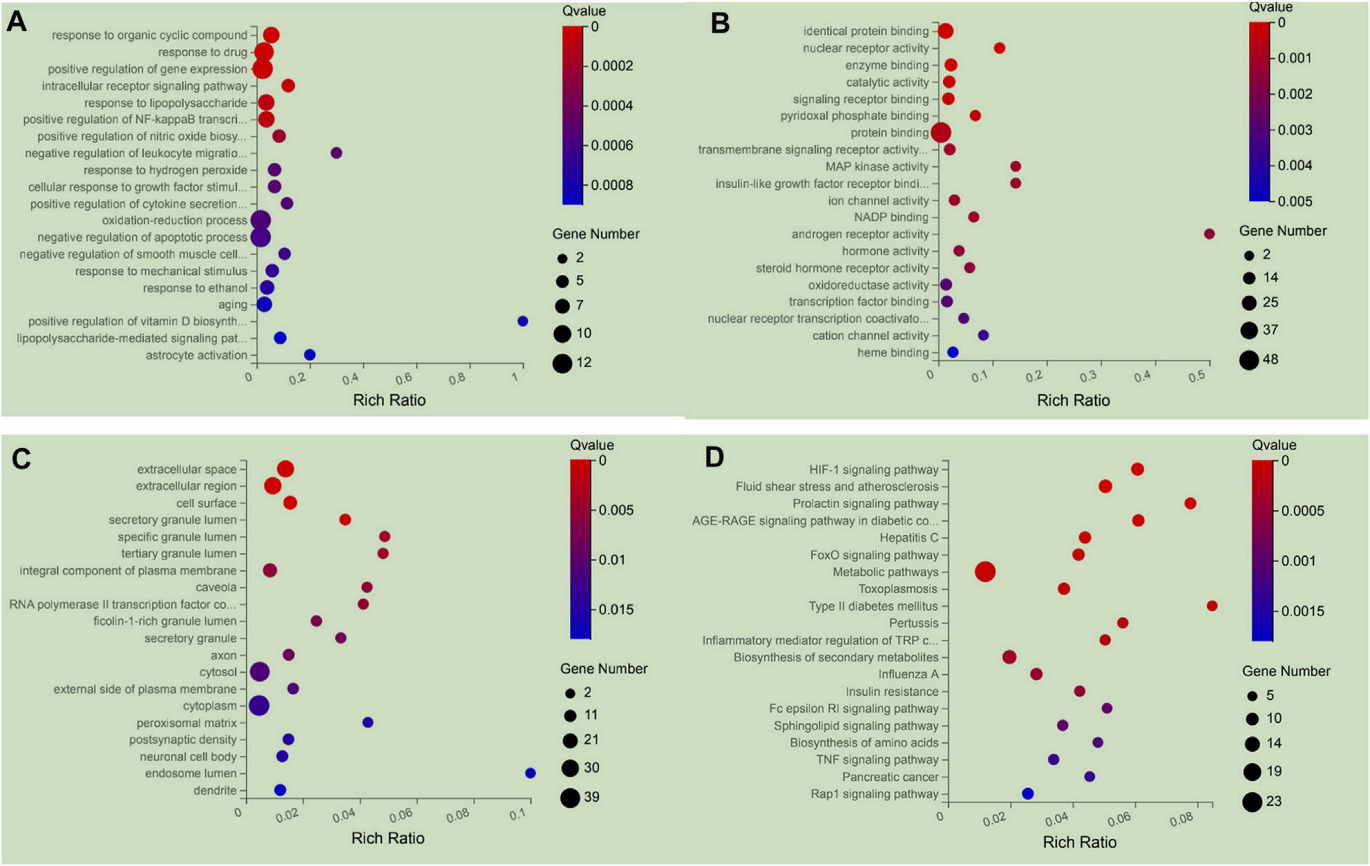
The 20 most significance therapy target genes of GO and KEGG pathway enrichment analysis of NFPX on ARDS. **(A)** GO analysis in biological process (BP); **(B)** GO analysis in molecular function (MF); **(C)** GO analysis in cellular component (CC). **(D)** KEGG pathway enrichment analysis.

To clarify the biological characteristics of putative targets of NFPX on ARDS in detail, the GO and KEGG pathway analyses of involved targets were conducted. The enrichment results included 1366 BP terms, 346 MF terms, and 188 CC terms. The top 20 significantly enriched terms in biological process (BP), molecular function (MF), and cellular component (CC) categories are shown in Figures 9A–C, which indicated that NFPX may regulate inflammatory action *via* identical protein binding, nuclear receptor activity and enzyme binding in extracellular space, extracellular region, and cell surface to exert its therapeutic effects on ARDS. 233 relevant pathways of NFPX were obtained by KEGG pathway enrichment. The key KEGG pathways of NFPX on ARDS are shown in Figure 9D, including the HIF-1 signaling pathway, AGE-RAGE signaling pathway, and FOXO signaling pathway, which are involved in the processes of oxidative stress, inflammatory response, cell metabolism, and cell cycle.

### RNA-Seq Analysis

**FIGURE 10:**
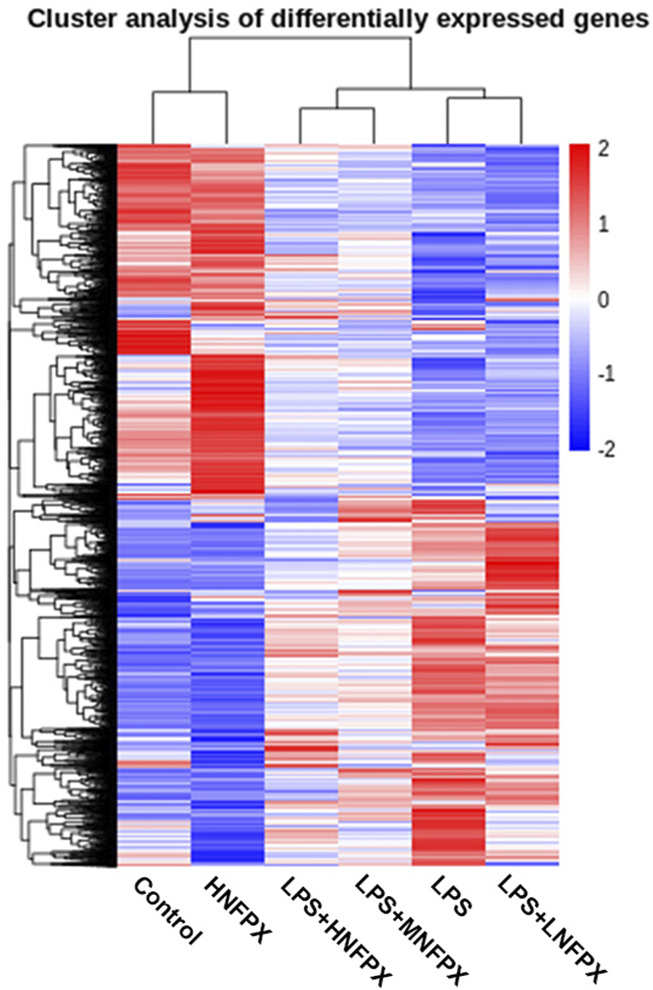
Hierarchical clustering analysis of genes that were differentially expressed in lung tissue samples using heatmap; each group contains four to five individuals. Blue-white indicates lower expression, and red indicates high expression.

To further verify the target genes, six groups of mice lung tissues were analyzed for RNA-seq detection. Overall, more than 1965 million reads were acquired and the percentages of uniquely mapped paired reads were 87.19–89.38%. Hierarchical clustering heatmap illustrated 11629 significantly DEGs. It is clear that such a cluster thermogram successfully separates the control group from the LPS group. In contrast, the gene expression profile of LPS+NFPX group lies between the control group and LPS group and the gene expression profile of the LPS+LNFPX group was more similar to that of the LPS group compared with that of the LPS+MNFPX group and LPS+HNFPX group (Figure 10).

**FIGURE 11:**
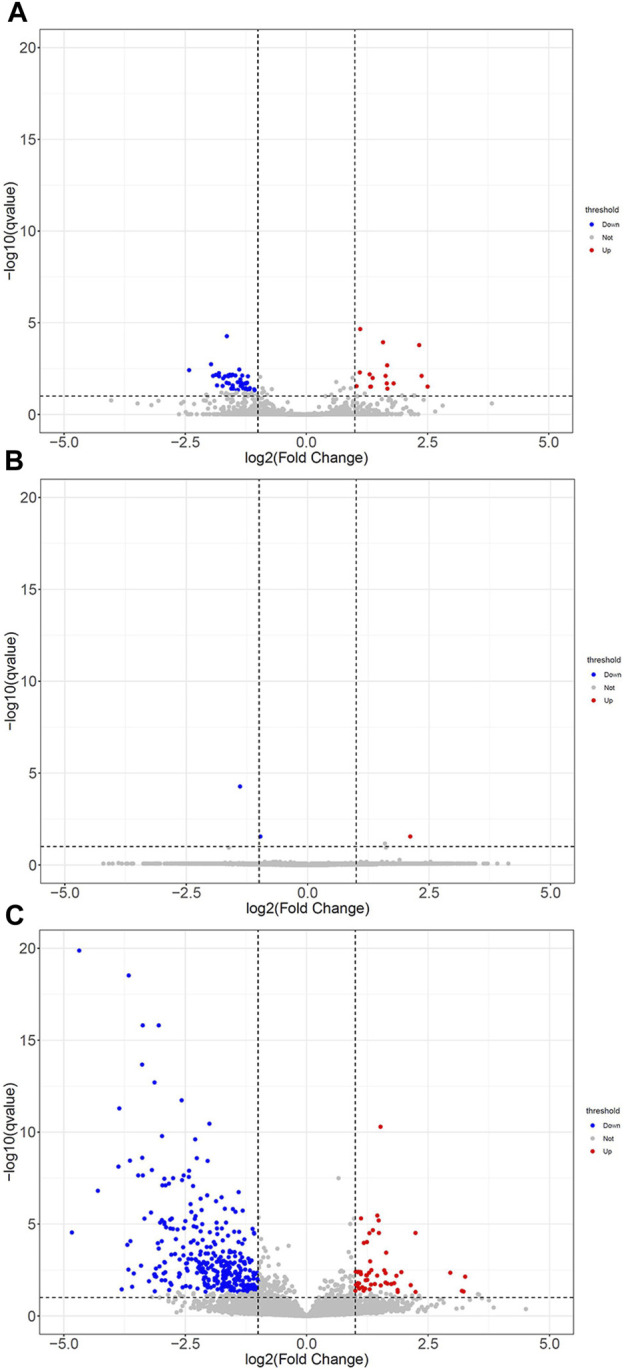
The DEGs with statistical significance from lung tissues between ARDS mice and ARDS mice were pretreated by different concentrations of NFPX screened using a volcano plot. Red notes indicate upregulated genes, and blue notes indicate downregulated genes. **(A)** LPS group vs. LPS+LNFPX group; **(B)** LPS group vs. LPS+MNFPX group; **(C)** LPS group vs. LPS+HNFPX group.

Then, these DEGs were further subjected to annotation with volcano maps by DESeq2 software. Compared with the LPS group, 21 significantly upregulated genes and 48 downregulated genes in the LPS+LNFPX group; one upregulated gene and two downregulated genes in the LPS+MNFPX group; 96 upregulated gene and 403 downregulated genes in the LPS+HNFPX group were screened (Figure 11). The summary of upregulated and downregulated genes is presented in Supplementary Table S6.

**FIGURE 12:**
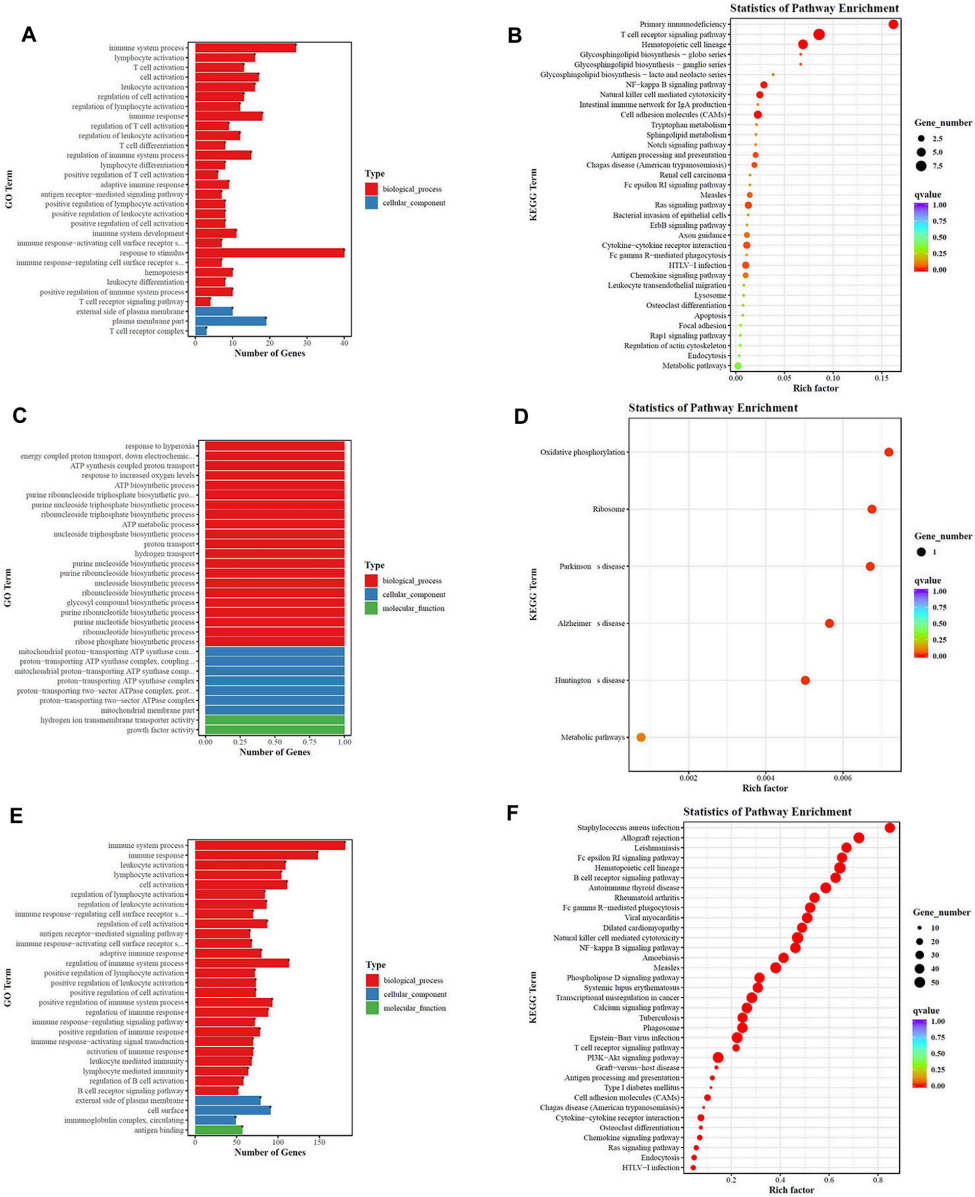
GO analysis **(A**,**C**,**E)** and KEGG pathway analysis **(B**,**D**,**F)** of the biological function of differentially regulated genes. **(A**,**B)** LPS group vs. LPS+LNFPX group; **(C**,**D)** LPS group vs. LPS+MNFPX group; **(E**,**F)** LPS group vs. LPS+HNFPX group.

At last, we performed GO and KEGG pathway analysis to highlight the up- and downregulation of four groupings of genes. As depicted in Figure 12, top 20 generally changed GO terms and KEGG pathways were ranked by enrichment score. The immune-inflammation response pathway had the largest number of DEGs. The most enriched GO terms of LPS vs. LPS+LNFPX included immune system process, lymphocyte activation, and T cell activation (Figure 12A). The mainly enriched GO terms of LPS vs. LPS+MNFPX included response to hyperoxia, energy coupled proton transport, and ATP synthesis (Figure 12C). The represented enriched GO terms of LPS vs. LPS+HNFPX included immune system process, immune response, and leukocyte activation (Figure 12E). Analogously, KEGG enrichment analysis also displayed that the mainly enriched pathways were connected with the immune-inflammation response. The most enriched KEGG pathways of LPS vs. LPS+LNFPX included primary immunodeficiency, T cell receptor signaling pathway, and hematopoietic cell lineage (Figure 12B). The most enriched KEGG pathways of LPS vs. LPS+MNFPX included oxidative phosphorylation, ribosome, and Parkinson’s disease (Figure 12D). The most enriched KEGG pathways of LPS vs. LPS+HNFPX included *Staphylococcus aureus* infection, allograft rejection, and Leishmaniasis (Figure 12F).

### Specific Gene Module–Based Target Identification for NFPX Based on the Transcriptional Data

We here utilized a gene module pair–based target identification (GMPTI) approach (http://www.bcxnfz.top/TMP/) to predict biological targets based on NFPX-induced gene expression profiles. GMPTI was proposed based on the assumption that similar drugs induced similar gene expression responses. Firstly, a specific transcriptional gene module pair (GMP) was automatically extracted for each target-induced transcriptional profile and can be used as a gene signature to represent the target. Then, for NFPX, we can calculate correlation scores for the GMPs of each target with the NFPX-induced gene expression profiles (see Methods). The correlation analysis among groups shown in Supplementary Table S7 suggests that the data are reliable. 3275 potential targets are listed in Supplementary Table S8 by comparing the *p* value of the LPS group with LPS+NFPX groups. With comprehensive analysis of network pharmacology, transcriptomics, and artificial intelligence, eight ARDS-related targets were selected: SMAD4, HIF-1, AMPK, HRAS, SOD1, AKT2, RAC1, and P53.

Then, these targets were docked by the NFPX ingredients with a three-dimensional structure on the representative conformations using the SYBYL − Surflex docking in standard precision mode. The docking results were ranked based on the CScore ranking (Supplementary Table S9). We observed many compound–target interactions with high docking scores. For example, with the docking score of 5 as the threshold, we can find that 63, 1, 105, 87, 84, 78, 99, and 17 ingredients interact with AKT2, AMPK, HARS, HIF-1, P35, RAC1, SMAD4, and SOD1, respectively. More specifically, with the docking score ranking, some potential active components of NFPX can be screened from these compounds. For example, the compound astragaloside IV interacts with SMAD4, P35, HIF-1, AKT2, RAC1, HARS, AMPK, and SOD1 with a docking score of 11.56, 10.86, 10.6, 9.75, 9.15, 8.94, 5.57, and 5.05, respectively, indicating that astragaloside IV may function by a multi-target mode. Similarly, neochlorogenic acid interacts with P35, HIF-1, RAC1, SMAD4, HARS, SOD1, AKT2, and AMPK with a docking score of 10.82, 9.92, 9.64, 8.87, 8.38, 7.71, 7.32, and 4.57, respectively.

### Confirmation of the Targets in ARDS Mice

**FIGURE 13:**
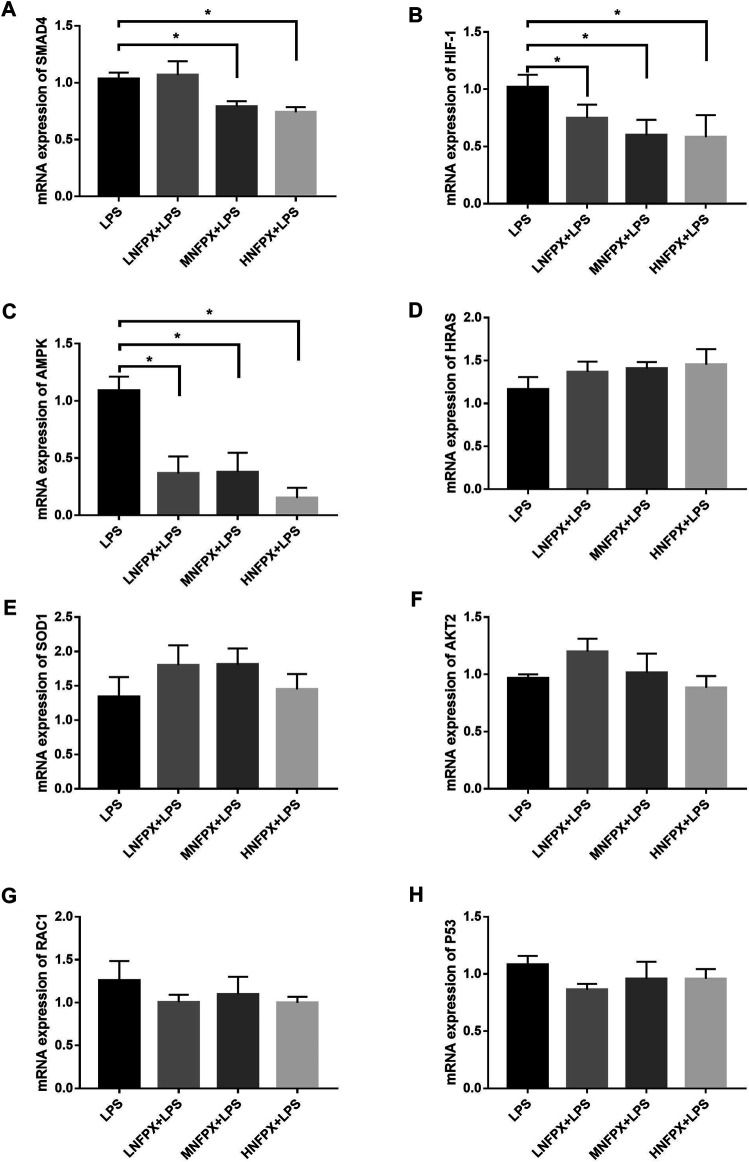
mRNA expression of potential targets of NFPX by qRT-PCR. *p < 0.05.

At last, the potential targets mentioned above were verified by qRT-PCR. As illustrated in Figure 13, SMAD4 expression was significantly downregulated in the MNFPX treated group and HNFPX treated group compared with LPS-treated group (*p* < 0.05) (Figure 13A). Moreover, the NFPX-treated group significantly decreased HIF-1 and AMPK expression in in a dose-dependent manner (*p* < 0.05) (Figures 13B,C). In contrast, there was no statistical difference in the expression of HRAS, SOD1, AKT2, RAC1, and P53 in NFPX+LPS groups when compared with LPS group (Figures 13D–H).

## Discussion

There has been a long history of using TCM in treating pulmonary diseases. However, the complexity of components of formula and ambiguity of mechanisms prevent their widespread use. In the previous literature, almost all studies about ARDS treatment by TCM have focused on the single component or bioactive molecules extracted from TCM (Li et al., 2018; Long et al., 2020). The appearance of network pharmacology analysis and high throughput sequencing break the barriers and greatly promote the development of TCM theory. Here, we demonstrated that NFPX can block the occurrence and development of ARDS for the first time. NFPX can alleviate lung impairment and prevent airway mucus overproduction *via* inhibiting cell apoptosis and inflammation, which coincide with the current treatment strategies of ARDS that modify the inflammatory process or promote the re-establishment of functional lung tissue. Furthermore, we explored the potential molecular mechanisms of NFPX against the ARDS by integrating network pharmacology, transcriptome analysis, and artificial intelligence analysis.

ARDS is a group of clinical disorders characterized by noncardiogenic pulmonary edema. Lung ultrasound examination has been widely used to evaluate pulmonary edema in intensive care units due to several advantages, including high sensitivity, bedside examination, no radiation, and real-time assessment. Nevertheless, it has been rarely reported that lung ultrasound was applied in the ARDS mice model because of their small size (Rubin et al., 2016). Here, an ultrahigh-frequency transducer probe was adopted to obtain high-resolution images. Our data gave preliminary evidences that NFPX relieved the alveolar interstitial syndrome and pleural thickening. Given the spatial heterogeneity of lung lesions in ARDS, both normal and abnormal artifacts can be observed in the same image. How to compare the scope, extent, types of lung lesions remains problematic. Therefore, further studies are needed for quantitative analysis of lung injury.

It is widely believed that inflammation response and oxidative stress are the most prominent initial causes of ARDS. We not only illustrated the therapeutic effect of NFPX in ARDS from the macroperspective by lung ultrasound but also investigated the influence of NFPX on pathomorphological changes, apoptosis, release of cytokines from local lung tissues, and blood circulation in the ARDS mouse model. The LPS intratracheal instillation mouse model is a reliable and reproducible mouse model of ARDS (Quijada et al., 2020). It has been widely used for fundamental research due to its similar pathophysiology to human ARDS. In our study, we observed that the degree of lung injury, lung W/D weight ratio, inflammatory cells infiltration, cell apoptosis, and cytokines release induced by LPS were significantly improved under the intervention of NFPX, and these effects manifested an apparent dose-dependent manner. Besides, apparent side effects were not observed in mice after 1 wk of HNFPX administration. Reportedly, as the main components of NFPX, Carthami Clos and Scutellariae Radix play an essential role in treating LPS-induced ARDS (Zhang et al., 2017; Long et al., 2020; Davis et al., 2021).

The above results have authenticated that NFPX played a critical role in treating ARDS. Nevertheless, the underlying mechanism is still indeterminate. In this study, we integrated the data based on network pharmacology, transcriptome, and artificial intelligence analysis. Bioactive components were identified meeting the criteria of OB ≥ 30% and DL index ≥0.18, which were regarded as pharmacokinetically active. Moreover, the herbs-compounds-targets network indicated that 77 target genes were closely associated with 37 bioactive components of NFPX. GO analysis indicated that NFPX may regulate inflammatory action *via* identical protein binding, nuclear receptor activity, and enzyme binding in extracellular space, extracellular region, and cell surface to exert its therapeutic effects on ARDS. A study by John et al. has confirmed that the extracellular location and cell surface of essential genes are quite significant (Kuchtey and Kuchtey, 2014). Besides, considering that most of the essential genes contribute to protein binding, nuclear receptor activity, and enzyme binding, it is quite reasonable to predict that the potential mechanisms of NFPX may involve multiple biological processes and molecular functions. Furthermore, KEGG enrichment shows that the principal signaling pathways participated in the process of treating ARDS by NFPX, including HIF-1 signaling pathway, AGE-RAGE signaling pathway, and FOXO signaling pathway. Numerous researches have proved that HIF-1, as a promoter of inflammation storm, could aggravate the inflammation and lung injury of ARDS (Suresh et al., 2019; Jahani et al., 2020; Serebrovska et al., 2020). Advanced glycation end products (AGE) could activate its receptor RAGE and promote oxidative stress leading to cell damage and inflammation (Shen et al., 2020). The roles of the AGE-RAGE signaling pathway are involved in lung diseases such as ARDS, lung cancer, and idiopathic pulmonary fibrosis and have been demonstrated in previous reports (Machahua et al., 2016; Ahmad et al., 2018; Zhu et al., 2021). Besides, Sandeep et al. have announced that Forkhead box-O (FOXO) is essential in the exudative phase of ARDS (Artham et al., 2019). In sum, the data of network pharmacology provide preliminary insights into the action mechanism of NFPX against ARDS.

In addition, we explored the underlying mechanism *via* transcriptome analysis. Consistent with the phenomena that NFPX mitigates lung edema, cell apoptosis, and inflammatory reaction induced by LPS in a dose-dependent manner, the gene clusters profile shown in the heatmap also manifested the same trend. Unexpectedly, only one upregulated gene and two downregulated genes in the LPS vs. LPS+MNFPX group were screened probably due to sequencing error affecting the data reliability of the LPS+MNFPX group in the following analysis. Despite that, the GO and KEGG enrichment pathway analysis in the LPS vs. LPS+LNFPX group and LPS+HNFPX group revealed that pathways associated with immune-inflammation response were the core regulation mechanism, which was in accordance with the previous data. What is more, SMAD4, HIF-1, and AMPK were screened by comprehensive analysis of network pharmacology, transcriptomics, and artificial intelligence. SMAD4, a member of the SMAD family of signal transduction proteins, is expressed in alveolar epithelial cells and has an inhibitory effect on tumors by reducing angiogenesis and increasing blood vessel hyperpermeability. However, Zhang and his colleagues have found that ARDS-associated pulmonary fibrosis might ameliorate through the SMAD4 signaling pathway (Zhang et al., 2015). It is well known that hypoxia is a key feature of ARDS accompanied by multiple important cellular processes, including cell apoptosis, inflammatory response, and angiogenesis regulated by HIF-1. Data from previous studies have confirmed the important effects of HIF-1 in ARDS (Harris et al., 2019; Suresh et al., 2019; Wang et al., 2020). Ample amounts of evidence support the idea that the AMPK pathway exerts its effects in LPS-induced ARDS (Wang et al., 2016; Bone et al., 2017; Chen et al., 2018). Therefore, the above evidence suggested that NFPX can effectively treat ARDS by regulating the gene expression level of SMAD4, HIF-1, and AMPK to interfere with the immune-inflammation response. Moreover, further validation experiments are required in the future.

## Conclusion

In conclusion, we prove the efficacy of NFPX decoction in the treatment of ARDS, thus rationalizing its potential as a novel therapeutic regime for ARDS treatment. Additionally, integrating network pharmacology, transcriptome, and artificial intelligence analysis illustrates the molecular mechanism of NFPX decoction on ARDS.

## Data Availability

The raw data supporting the conclusions of this article will be made available by the authors, without undue reservation.
