# Supplementary material for: Integrating Network Pharmacology, Transcriptome and Artificial Intelligence for Investigating Into the Effect and Mechanism of Ning Fei Ping Xue Decoction Against the Acute Respiratory Distress Syndrome
Source: Front Pharmacol. 2021 Nov 3;12:731377. doi: 10.3389/fphar.2021.731377 (PMC8595141; doi:10.3389/fphar.2021.731377)
Supplement: Supplementary file 3 [file Table1.DOCX]

| No. | Chinese Name | English Name | Structural Formula |
| --- | --- | --- | --- |
| 1 | 腺苷 | Adenosine | 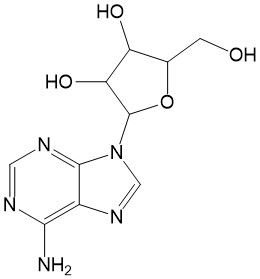 |
| 2 | 梓醇 | Catalpol | 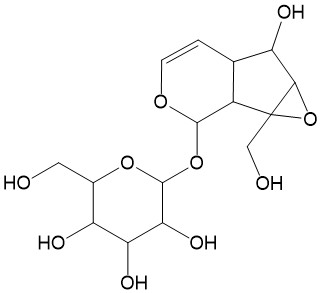 |
| 3 | 鸟苷 | Guanosine | 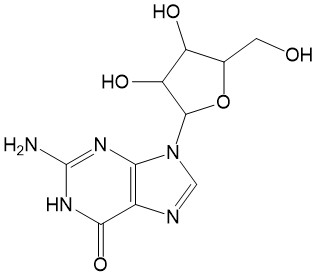 |
| 4 | 果糖苯丙氨酸 | Fructose-phenylalanine | 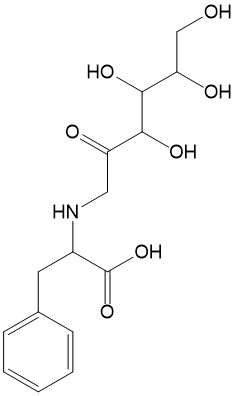 |
| 5 | 地黄苷 D | Rhmannioside D | 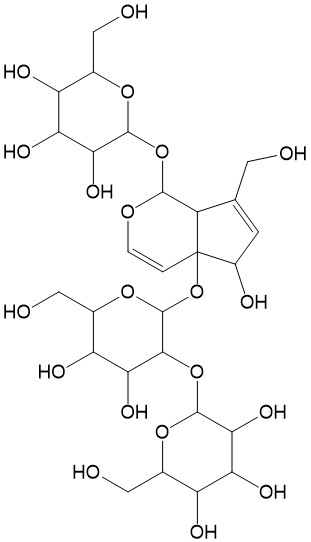 |
| 6 | 灯盏花苷C | Erigeside C | 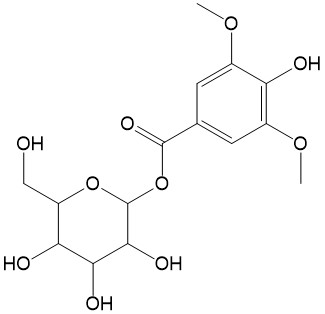 |
| 7 | / | O-β-D-Gentiobiosyl-D-(-)-mandelamide | 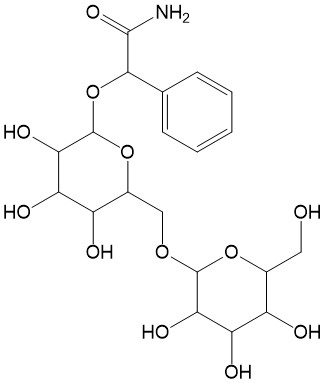 |
| 8 | L-色氨酸 | L-Tryptophan | 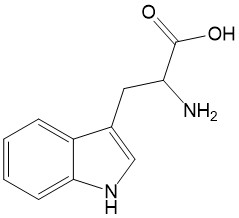 |
| 9 | 山栀子苷 | Shanzhiside | 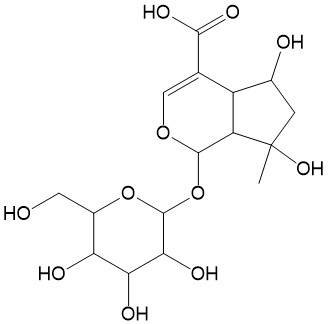 |
| 10 | 鸡矢藤次苷甲酯 | Feretoside | 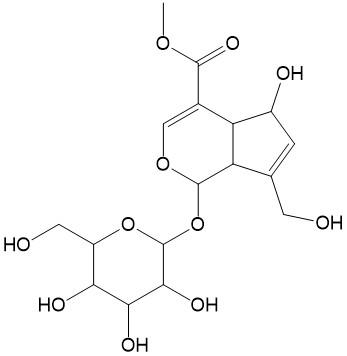 |
| 11 | / | 5-​Deoxylamiol | 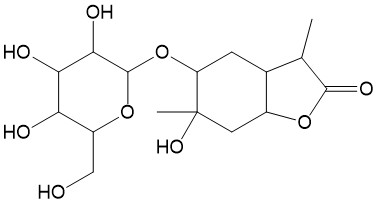 |
| 12 | 木兰箭毒碱 | Magnocurarine | 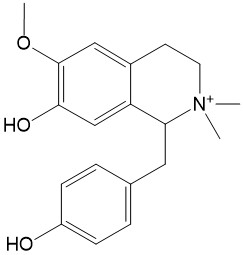 |
| 13 | 羟异栀子苷 | Gardenoside | 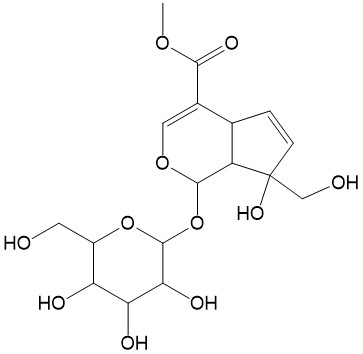 |
| 14 | / | Succinyladenosine | 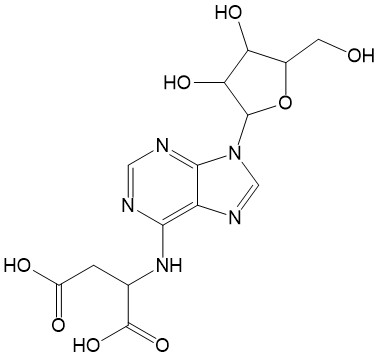 |
| 15 | / | L-(+)-mandelic acid-O-β-D-Gentiobioside | 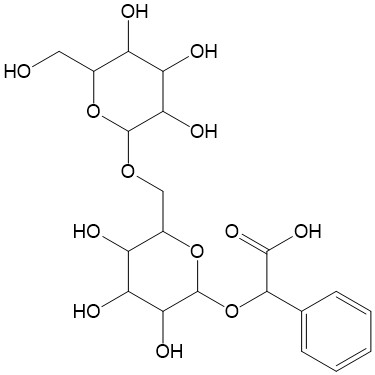 |
| 16 | 去乙酰车叶草苷酸甲酯 | Deacetyl asperulosidic acid methyl ester | 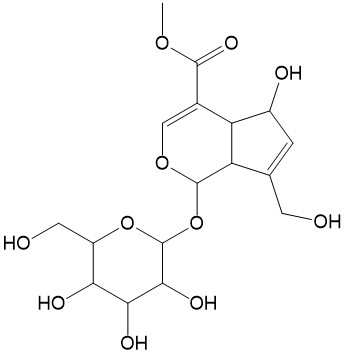 |
| 17 | / | Jasminoside B | 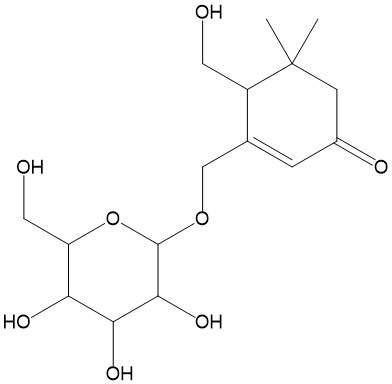 |
| 18 | 黄柏碱 | Phellodendrine | 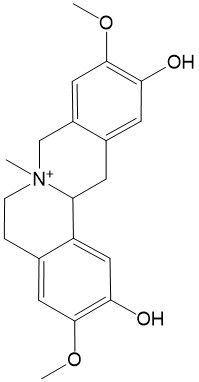 |
| 19 | / | D-(+)-mandelic acid-O-β-D-Gentiobioside |  |
| 20 | / | L-Phenylalaninosecologanin B | 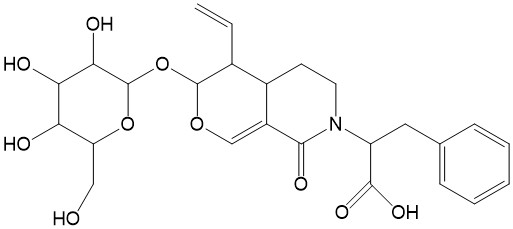 |
| 21 | 苍术苷A | Atractyloside A | 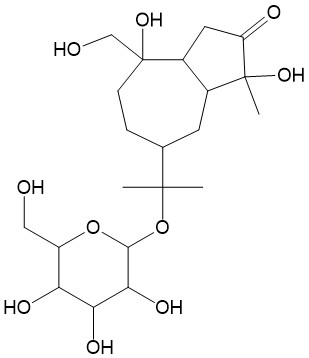 |
| 22 | / | Jasminoside D | 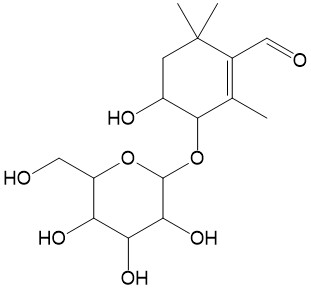 |
| 23 | 新绿原酸 | Neochlorogenic acid | 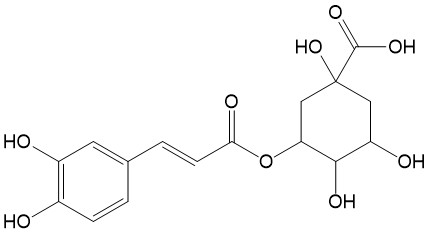 |
| 24 | N-甲基乌药碱 7-吡喃葡萄糖苷 | N-Methylhigenamine 7-glucopyranoside | 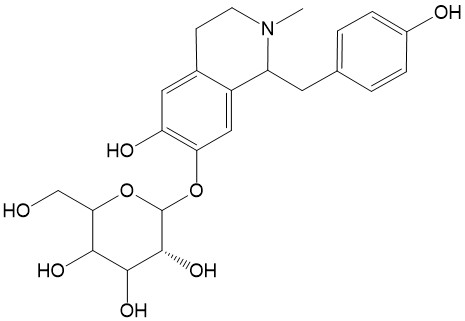 |
| 25 | 藤泊它碱 | Tembetarine | 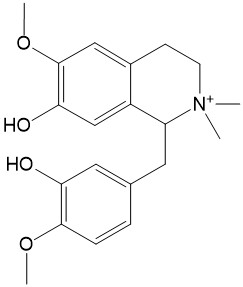 |
| 26 | 木兰花碱 | Magnoflorine | 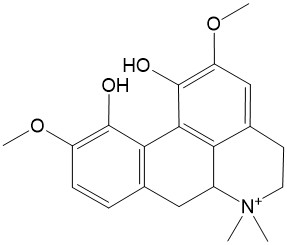 |
| 27 | 8-O-乙酰基玉叶金花苷 | 8-O-Acetylmussaenoside | 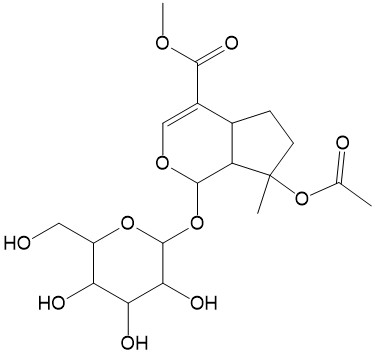 |
| 28 | / | Cuchiloside | 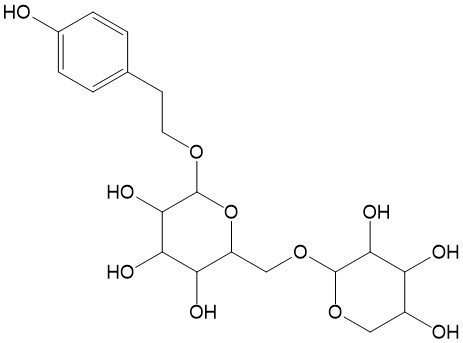 |
| 29 | 槲皮素-3-O-β-D-吡喃葡萄糖基-7-O-β-龙胆二糖苷 | Quercetin­3­-O­-β-­D­glucopyranosyl­-7­-O­-β­gentiobioside | 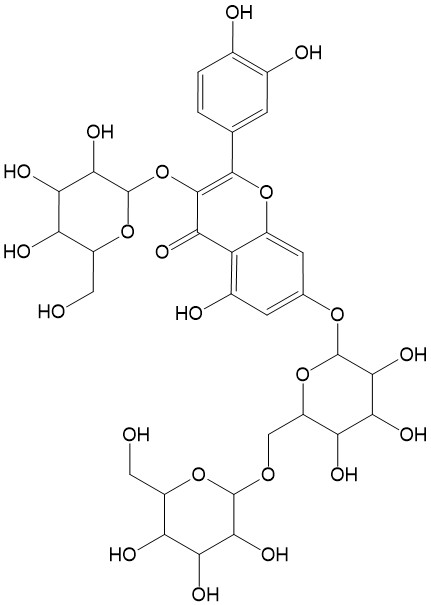 |
| 30 | L-苦杏仁苷 | L-Amygdalin | 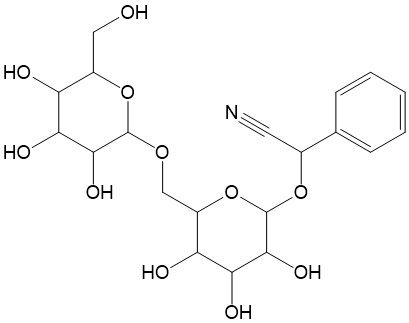 |
| 31 | / | Oblongine | 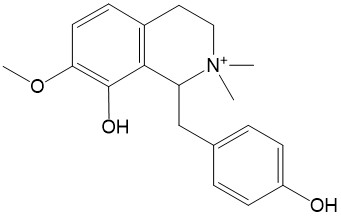 |
| 32 | D-苦杏仁苷 | D-Amygdalin | 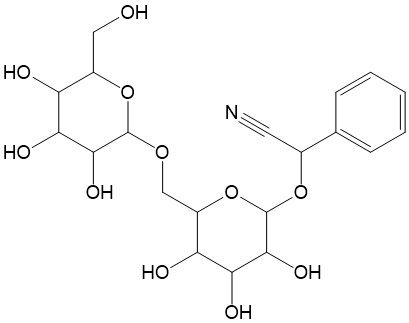 |
| 33 | 京尼平1-龙胆双糖苷 | Genipin 1-gentiobioside | 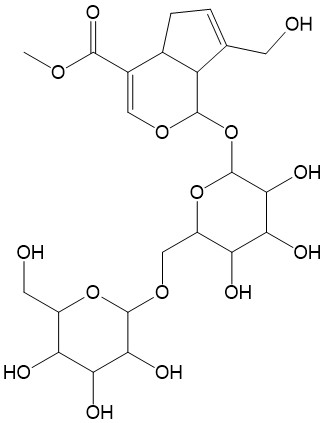 |
| 34 | / | Lycoranine B | 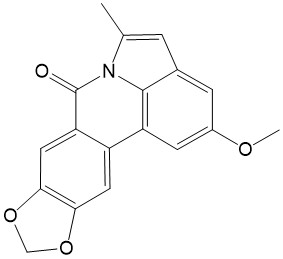 |
| 35 | 蝙蝠葛任碱 | Menisperine | 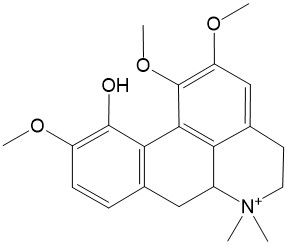 |
| 36 | 绿原酸 | Chlorogenic acid | 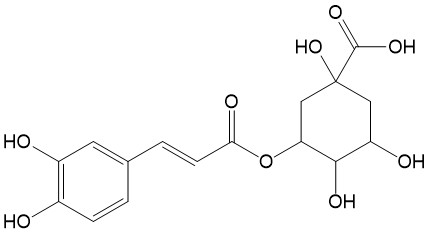 |
| 37 | 京尼平苷 | Geniposide | 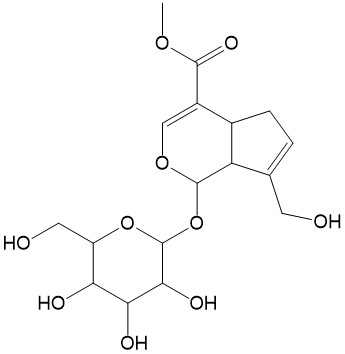 |
| 38 | / | (13aS)-5,8,13,13a-Tetrahydro-3,9,10-trimethoxy-6H-dibenzo[a,g]quinolizin-2-yl β-D-glucopyranoside | 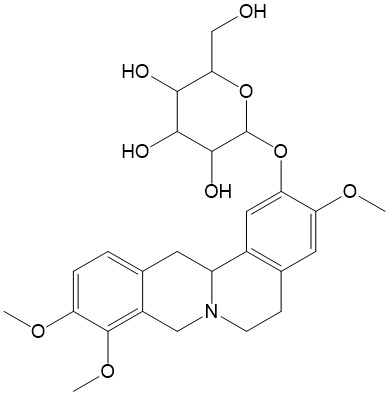 |
| 39 | 5-O-阿魏酰奎宁酸 | 5-O-Feruloylquinic acid | 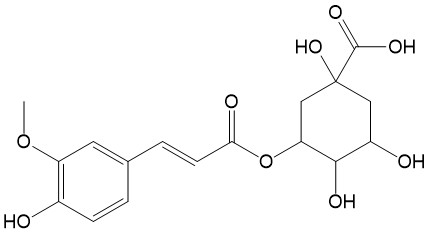 |
| 40 | 隐绿原酸 | Cryptochlorogenic acid | 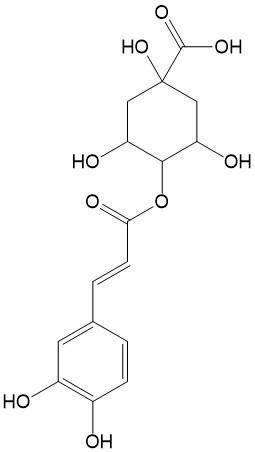 |
| 41 | 维生素B2 | Vitamin B2 | 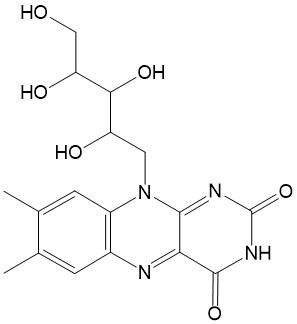 |
| 42 | 芍药内酯苷 | Albiflorin | 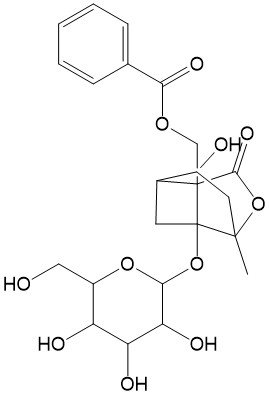 |
| 43 | / | 5,6,6a,7-Tetrahydro-11-hydroxy-1,2,10-trimethoxy-6,6-dimethyl-4H-dibenzo[de,g]quinolinium | 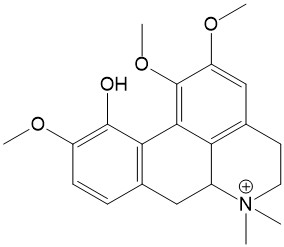 |
| 44 | / | Epijasminoside A | 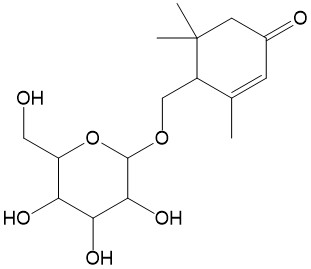 |
| 45 | 苦番红花素 | Picrocrocin | 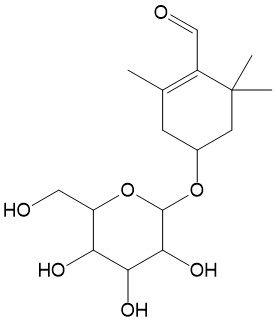 |
| 46 | 芍药苷 | Paeoniflorin | 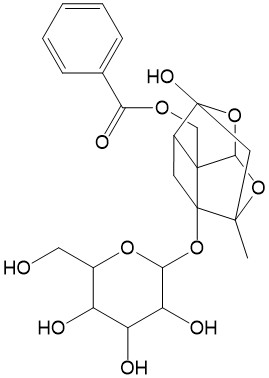 |
| 47 | 四氢巴马汀 | Tetrahydropalmatine | 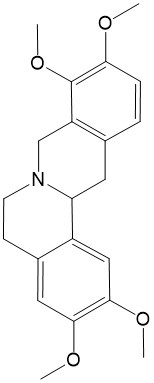 |
| 48 | 8-氧化表小檗碱 | 8-Oxoepiberberine | 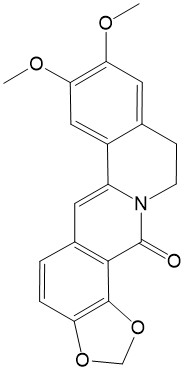 |
| 49 | 格兰地新 | Groenlandicine | 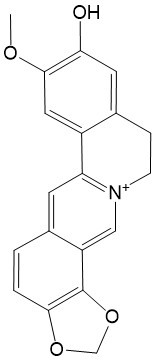 |
| 50 | 去亚甲基小檗碱 | Demethyleneberberine | 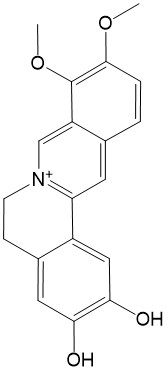 |
| 51 | 羟基红花黄色素A | Hydroxysafflor Yellow A | 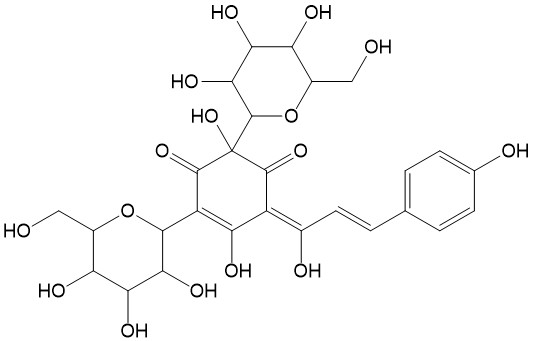 |
| 52 | 3-O-阿魏酰奎宁酸 | 3-O-Feruloylquinic acid | 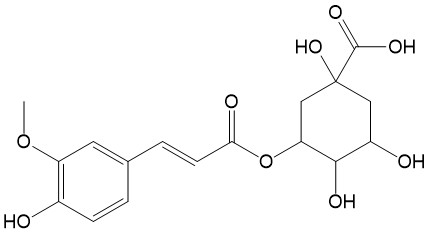 |
| 53 | / | Platydesmine | 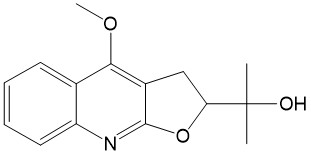 |
| 54 | / | Hyemaloside B | 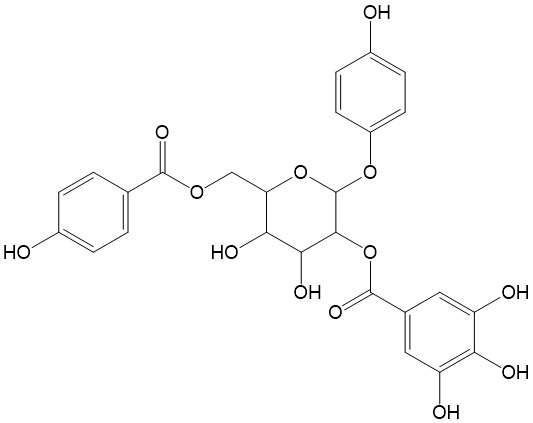 |
| 55 | N-甲基紫堇定 | N-Methylcorydine | 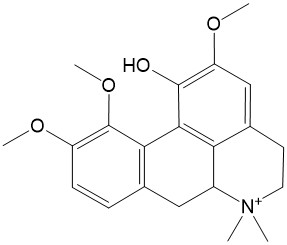 |
| 56 | 槲皮素3-O-葡萄糖基-芸香糖苷 | Quercetin 3-O-glucosyl-rutinoside | 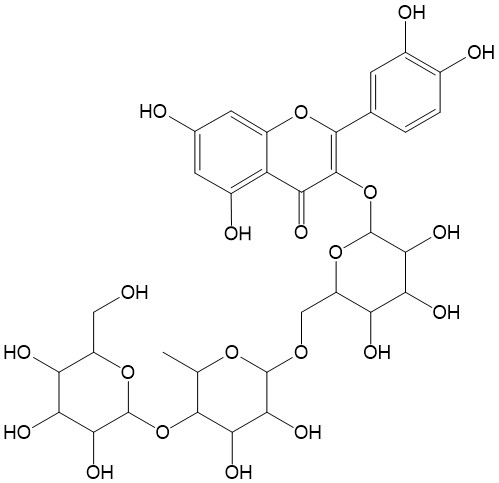 |
| 57 | N-甲基氢化小檗碱 | N-Methylcanadine | 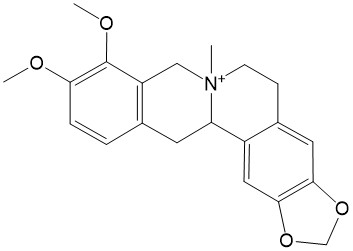 |
| 58 | 槲皮素7-O-β-龙胆二糖苷 | Quercetin­7­-O-­β­-gentiobioside | 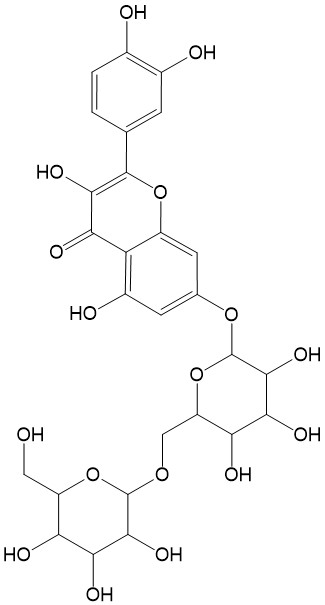 |
| 59 | 洋地黄叶苷 C | Purpureaside C | 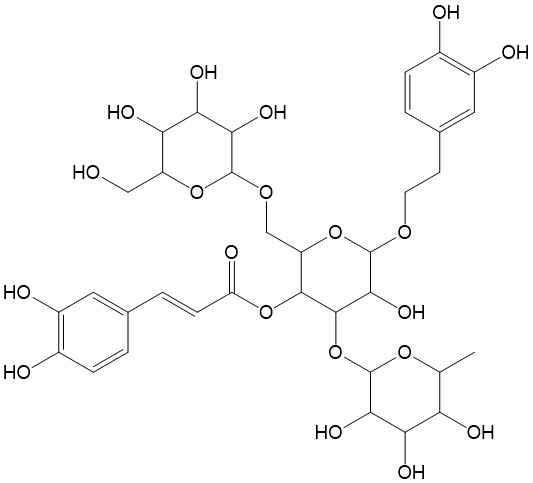 |
| 60 | 黄连碱 | Coptisine | 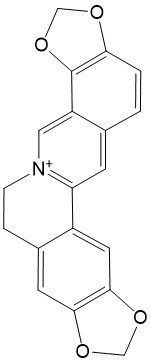 |
| 61 | 4-O-阿魏酰奎宁酸 | 4-O-Feruloylquinic acid | 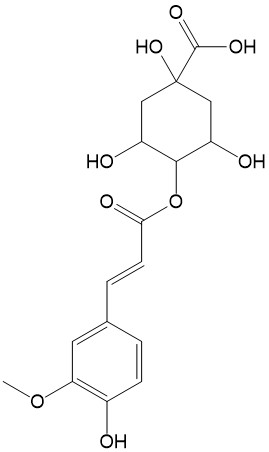 |
| 62 | 表小檗碱 | Epiberberine | 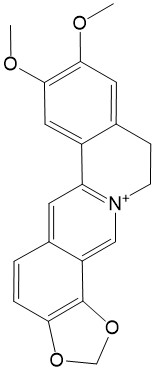 |
| 63 | / | 2-​hydroxyethyl, 6-​[(2E)​-​3-​(3,​4-​dihydroxyphenyl)​-​2-​propenoate]-β-​D-​Glucopyranoside | 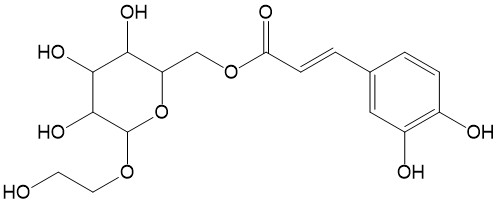 |
| 64 | 阿魏酸 | Ferulic Acid | 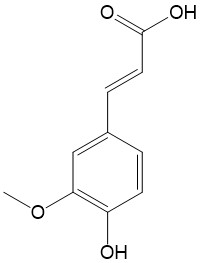 |
| 65 | 非洲防己碱 | Columbamine | 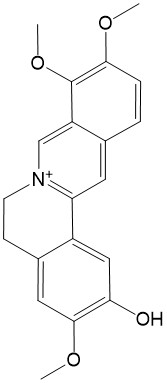 |
| 66 | 毛蕊异黄酮苷 | Calycosin-7-glucoside | 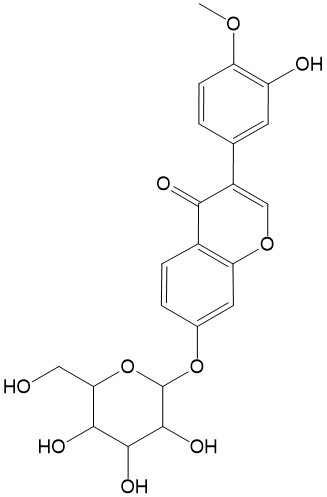 |
| 67 | 焦地黄苯乙醇苷A1 | Jionoside A1 | 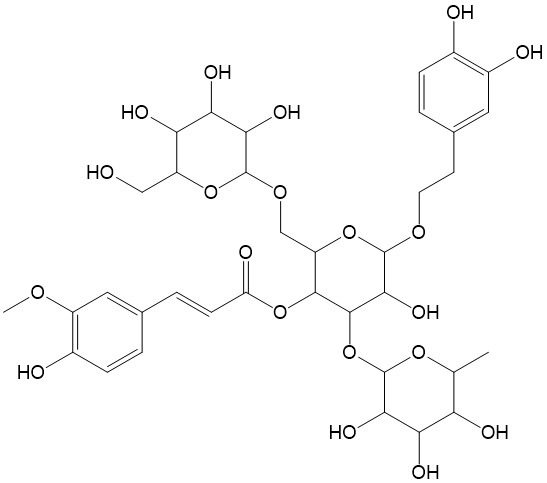 |
| 68 | 白杨素-6-C-六碳糖苷-8-C-五碳糖苷 | Chrysin-6-C-hexoside -8-C- pentoside | 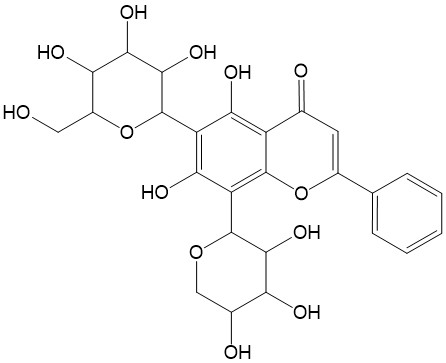 |
| 69 | 药根碱 | Jateorhizine | 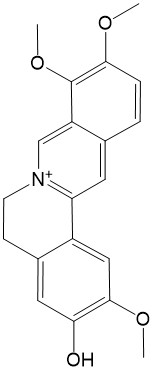 |
| 70 | 白杨素6-C-葡萄糖苷-8-C阿拉伯糖苷 | Chrysin-6-C-glucoside-8-C-arabinoside | 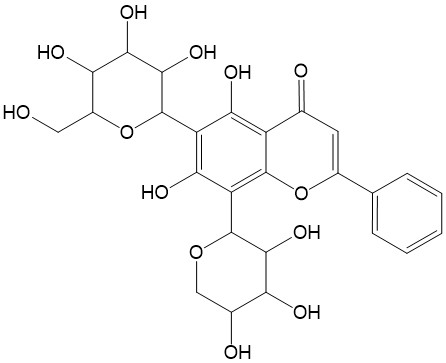 |
| 71 | 白杨素-6-C-六碳糖苷-8-C-五碳糖苷 | Chrysin-6-C-hexoside -8-C- pentoside |  |
| 72 | 氧化小檗碱 | Oxoberberine | 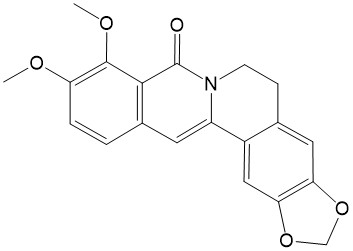 |
| 73 | 白杨素6-C-五碳糖苷-8-C六碳糖苷 | Chrysin-6-C-pentoside-8-C-hexoside | 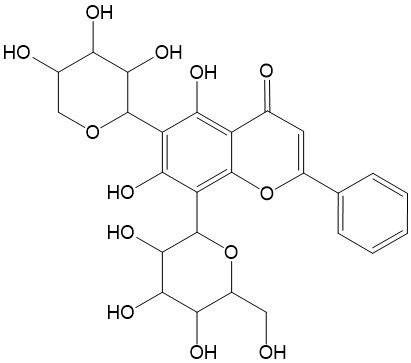 |
| 74 | 没食子酰芍药苷 | Galloylpaeoniflorin | 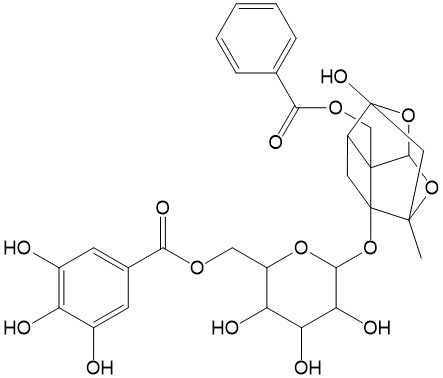 |
| 75 | 白杨素6-C-阿拉伯糖苷-8-C葡萄糖苷 | Chrysin-6-C-arabinoside-8-C-glucoside | 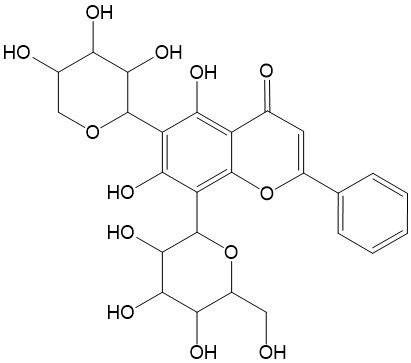 |
| 76 | 芦丁 | Rutin | 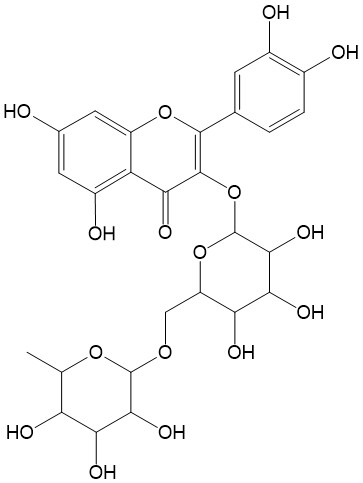 |
| 77 | 毛蕊花糖苷 | Aceteoside | 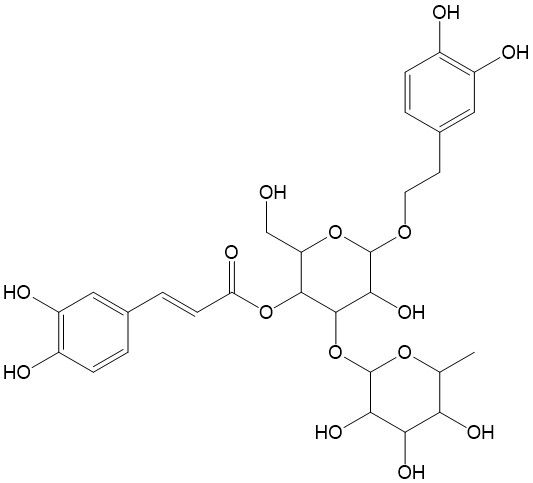 |
| 78 | 小檗碱 | Berberine | 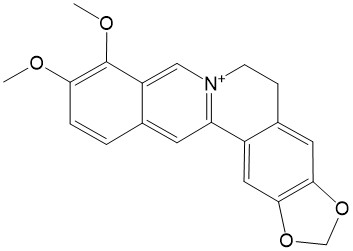 |
| 79 | 白杨素6-C-五碳糖苷-8-C六碳糖苷 | Chrysin-6-C-pentoside-8-C-hexoside |  |
| 80 | 巴马丁 | Palmatine | 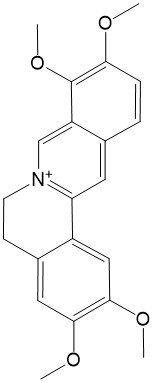 |
| 81 | 黄柏苷 | Amurensin | 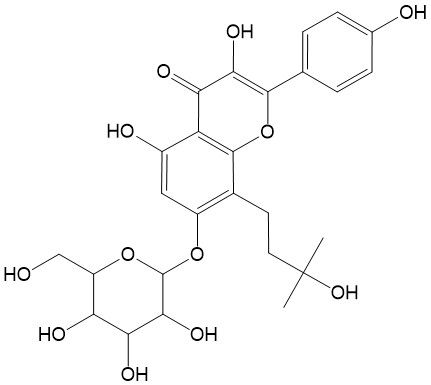 |
| 82 | / | Isomucronulatol-7,2'-di-O-glucoside | 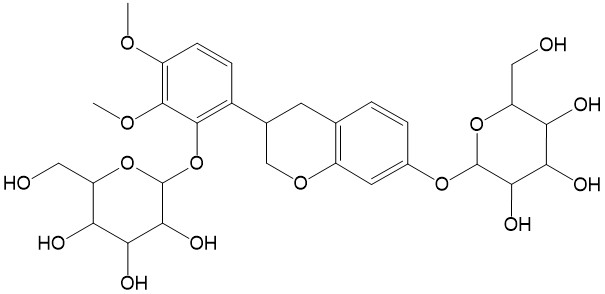 |
| 83 | 异毛蕊花糖苷 | Isoacteoside | 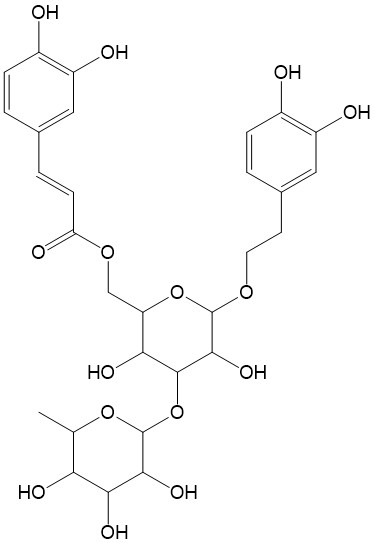 |
| 84 | 野黄芩苷 | Scutellarin | 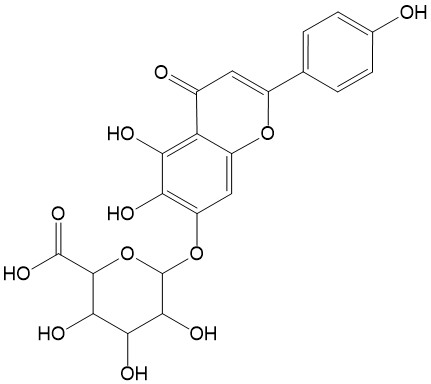 |
| 85 | / | Jasminoside I/H/S | 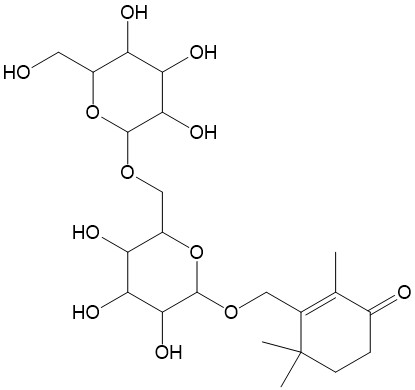 |
| 86 | 泽兰苷 | Eupatolin | 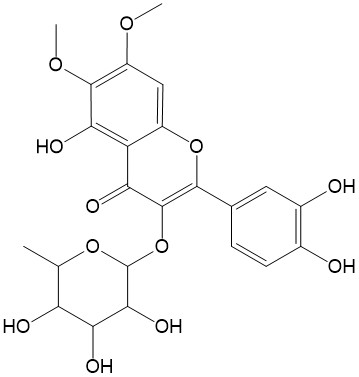 |
| 87 | 洋川芎内酯F | Senkyunolide F | 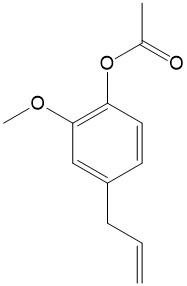 |
| 88 | 红车轴草素-7-O-D-葡萄糖苷 | Pratensein-7-O-glucoside | 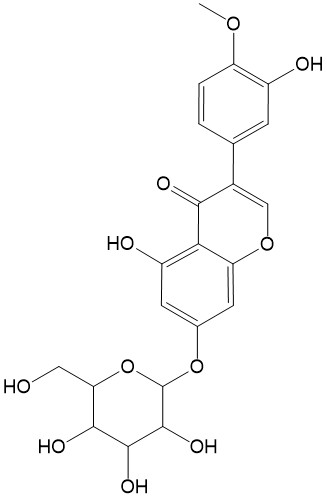 |
| 89 | 山奈酚-3-O-芸香糖苷 | Kaempferol-3-O-rutinoside | 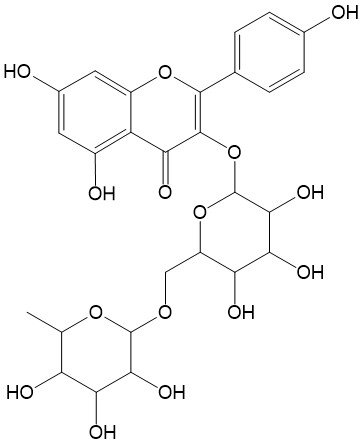 |
| 90 | 花椒宁碱 | Fagaronine | 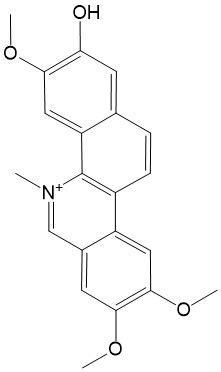 |
| 91 | 芍药新苷 | Lactiflorin | 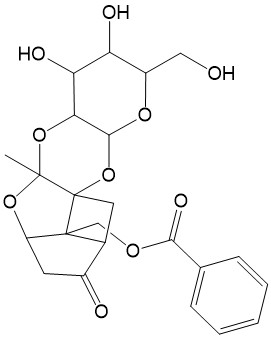 |
| 92 | 6''-O-[反式-介子酰]-京尼平龙胆双糖苷 | 6''-O-[trans-Sinapoyl] -genipin gentiobioside | 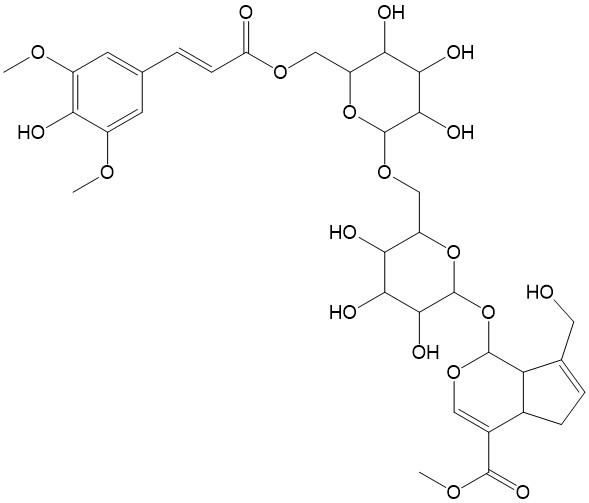 |
| 93 | 6''-O-[反式-阿魏酰]-京尼平龙胆双糖苷 | 6''-O-[trans-Feruloyl] genipin gentiobioside | 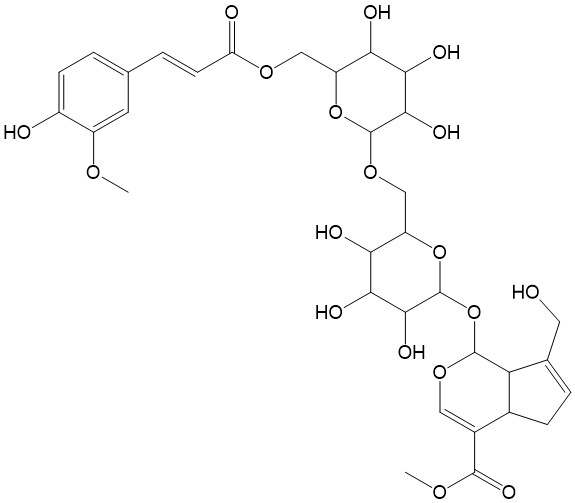 |
| 94 | 芒柄花苷 | Ononin | 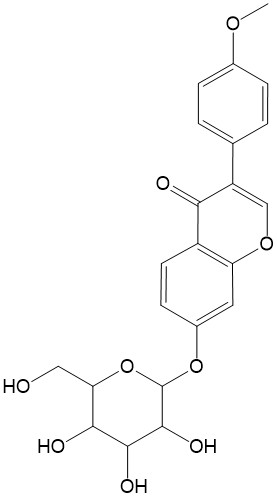 |
| 95 | 西红花苷Ⅰ | Crocin I | 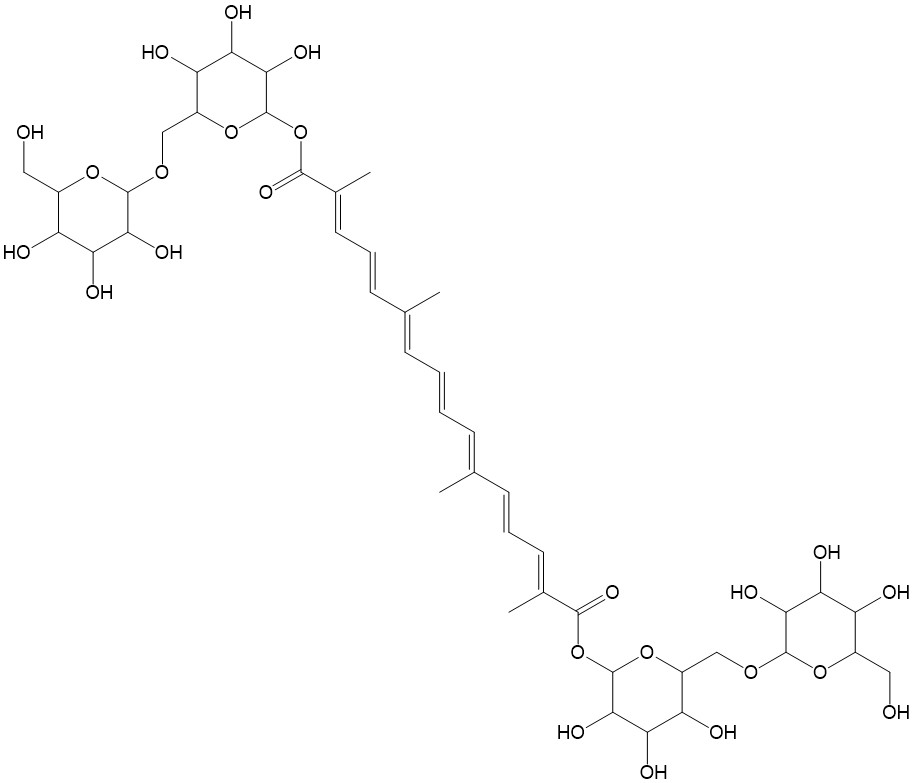 |
| 96 | / | 6’-O-trans-Sinapoyljasminoside L | 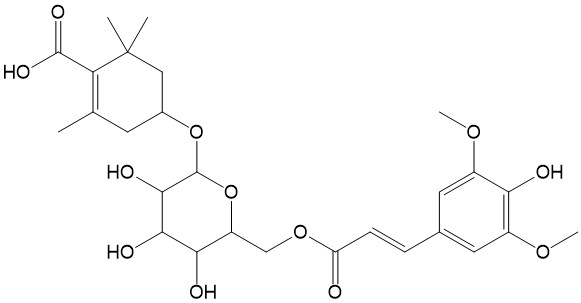 |
| 97 | 芹菜素-7-O-β-D-葡萄糖苷 | Apigenin-7-O- β-D-glucoside | 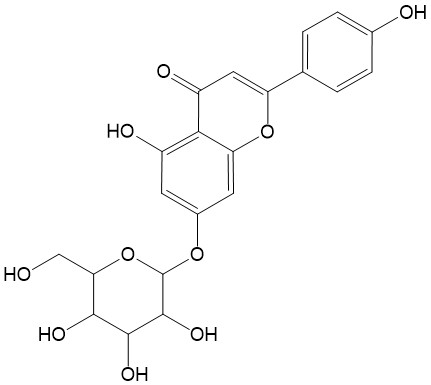 |
| 98 | 3-O-介子酰-5-O-咖啡酰奎宁酸 | 3-O-Sinapoyl-5-O-caffeoylquinic acid | 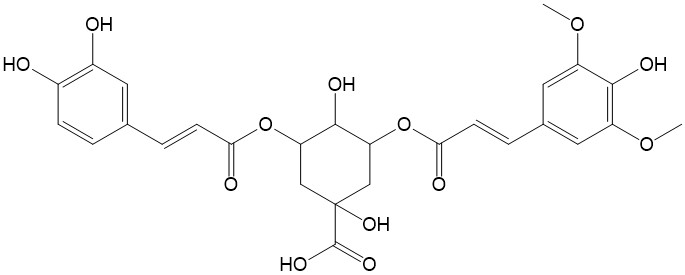 |
| 99 | / | 6’-O-trans-Sinapoyljasminoside L Isomer |  |
| 100 | 粘毛黄芩素III | Viscidulin III | 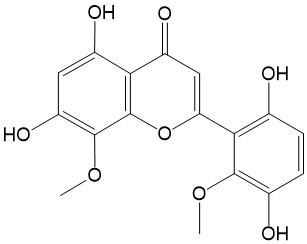 |
| 101 | 6'-O-芥子酰栀子苷 | 6'-O-sinapoylgeniposide | 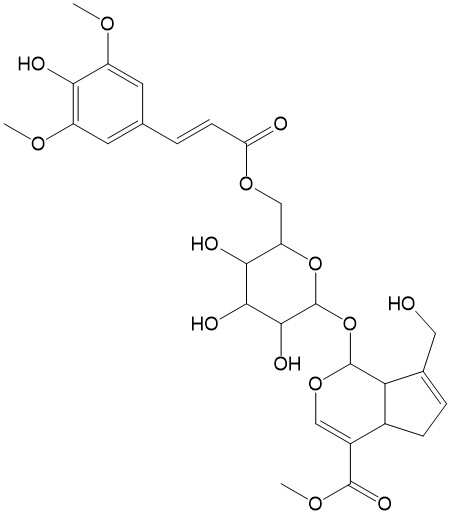 |
| 102 | 5,7,2'-三羟基-6-甲氧基黄酮-7-O-葡萄糖醛酸苷 | 5,7,2'-Trihydroxy-6-methoxy flavone-7-*O-*glucuronide | 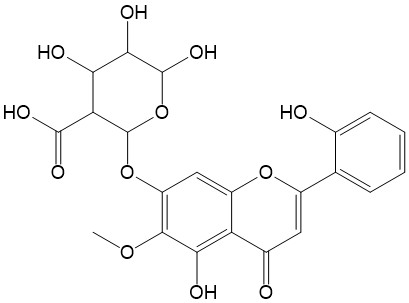 |
| 103 | 美迪紫檀苷 | Methylnissolin 3-O-glucoside | 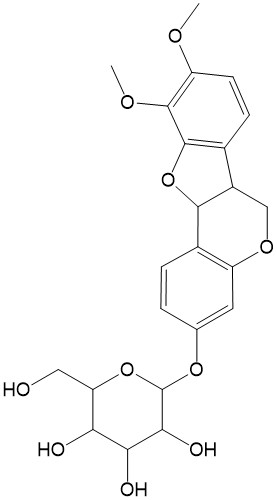 |
| 104 | 黄芩苷 | Baicalin | 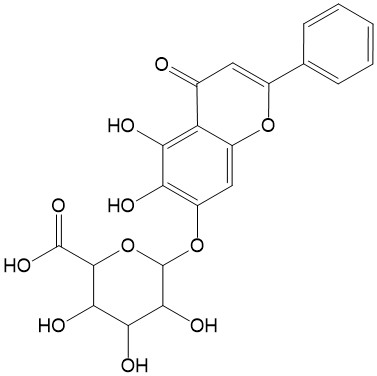 |
| 105 | 二氢黄芩苷 | Dihydrobaicalin |  |
| 106 | 4-O-介子酰-5-O-咖啡酰奎宁酸 | 4-O-sinapoyl-5-O-caffeoylquinic acid |  |
| 107 | 黄芪异黄烷苷 | Isomucronulatol-7-O-glucoside |  |
| 108 | 柚皮素-7-O-葡萄糖醛酸苷 | Naringenin-7-O-glucuronide |  |
| 109 | 3,5-二咖啡酰基-4-O-(3-羟基-3-甲基戊二酰基)奎宁酸 | 3,5-Di-O-caffeoyl-4-O-(3-hydroxy-3-methyl) glutaroylquinic acid |  |
| 110 | 3-O-介子酰-4-O-咖啡酰奎宁酸 | 3-O-sinapoyl-4-O-caffeoylquinic acid |  |
| 111 | 毛蕊异黄酮 | Calycosin |  |
| 112 | 去甲汉黄芩素7-O-葡萄糖醛酸苷 | Norwogonin 7-O-β-D-glucuronide |  |
| 113 | 香叶木素7-O-β-D-葡萄糖醛酸苷 | Diosmetin 7-O-β-D-glucuronide |  |
| 114 | 黄芩素 6-O-葡萄糖醛酸苷 | Baicalein 6-O-β-D-glucuronide |  |
| 115 | 白杨素-7-O-β-D葡萄糖醛酸苷 | Chrysin7-O-β-D-glucuronide |  |
| 116 | 千层纸素A 7-O-葡萄糖醛酸 | Oroxylin A 7-O-glucuronide |  |
| 117 | 5,6,7,-三羟基-8-甲氧基黄酮-7-葡糖醛酸吡喃糖苷 | 5,6,7-Trihydroxy-8-methoxyflavone-7-O-glucuronopyranoside |  |
| 118 | 汉黄芩苷 | Wogonoside |  |
| 119 | 黄芪皂苷VI | Astragaloside VI |  |
| 120 | 香叶木素 | 3',5,7-Trihydroxy-4'-methoxyflavone |  |
| 121 | 正丁基苯酞 | 3-N-butylphthalide |  |
| 122 | 西红花苷Ⅰ 异构体 | Crocin I |  |
| 123 | 黄芩素 | Baicalein |  |
| 124 | 黄芪皂苷VI 异构体 | Astragaloside VI Isomer |  |
| 125 | / | Pinellic acid |  |
| 126 | 黄芪甲苷 | Astragaloside IV |  |
| 127 | 16-氧泽泻醇A | 16-oxoalisol A |  |
| 128 | 西红花苷 Ⅲ | Crocin Ⅲ |  |
| 129 | 柠檬苦素 | Limonin |  |
| 130 | 黄芪皂苷Ⅱ | Astragaloside Ⅱ |  |
| 131 | 西红花苷 Ⅲ 异构体 | Crocin Ⅲ Isomer |  |
| 132 | 24-乙酰 16-氧化泽泻醇 A | 24-Acetyl 16-oxoalisol A |  |
| 133 | 异黄芪皂苷Ⅱ | Isoastragaloside Ⅱ |  |
| 134 | 汉黄芩素 | Wogonin |  |
| 135 | / | Cyclosiversioside D |  |
| 136 | 黄芩黄酮II | Skullcapflavone II |  |
| 137 | 洋川芎内酯A | Senkyunolide A |  |
| 138 | 千层纸素A | Oroxylin A |  |
| 139 | 白术内酯 III | Atractylenolide III |  |
| 140 | 泽泻醇C | Alisol C |  |
| 141 | 黄芪皂苷I | Astragaloside I |  |
| 142 | 12,13-二羟基-9Z,15Z-十八碳二烯酸 | 12,13-dihydroxy-9Z,15Z-octadecadienoic acid |  |
| 143 | 异黄芪皂苷I | Isoastragaloside I |  |
| 144 | 泽泻醇P | Alisol P |  |
| 145 | 新黄芪皂苷 I | Neoastragaloside I |  |
| 146 | 藁本内酯 | Ligustilide |  |
| 147 | 23-乙酰泽泻醇 C | 23-Acetyl alisol C |  |
| 148 | 白术内酯 II | Atractylenolide II |  |
| 149 | 泽泻醇A | Alisol A |  |
| 150 | 23-乙酰泽泻醇 B | 23-Acetyl alisol B |  |
